# Serine metabolism reprogramming in cancer: a multi-tiered regulatory framework: Serine metabolism reprogramming in cancer

**DOI:** 10.3724/abbs.2025188

**Published:** 2025-11-11

**Authors:** Yi Yuan, Keru Wang, Yuxin Jin, Tianyu Han

**Affiliations:** 1 Jiangxi Institute of Respiratory Disease the Department of Respiratory and Critical Care Medicine the First Affiliated Hospital Jiangxi Medical College Nanchang University Nanchang 330052 China; 2 School of Huankui Academy Nanchang University Nanchang 330031 China; 3 Jiangxi Clinical Research Center for Respiratory Diseases Nanchang 330006 China; 4 China-Japan Friendship Jiangxi Hospital National Regional Center for Respiratory Medicine Nanchang 330200 China; 5 The MOE Basic Research and Innovation Center for the Targeted Therapeutics of Solid Tumors Jiangxi Medical College Nanchang University Nanchang 330031 China

**Keywords:** serine metabolism, serine metabolic enzymes, transcriptional regulation, transcription factor, post-transcriptional regulation, non-coding RNAs, post-translational modifications

## Abstract

As a critical component of amino acid metabolic reprogramming, serine metabolism has been demonstrated to be enhanced in a variety of cancer types, thereby supporting tumor progression. This enhancement is primarily driven by increased expression levels and augmented enzymatic activity of serine metabolic enzymes (phosphoglycerate dehydrogenase, phosphoserine aminotransferase 1, phosphoserine phosphatase and serine hydroxymethyltransferase). However, there is still lack of comprehensive summary on the regulation of serine metabolism in cancer. In this review, we provide a systematic overview of the currently discovered and proven regulatory mechanisms of serine metabolic enzymes in cancer, focusing on three levels: transcriptional, post-transcriptional, and post-translational regulation. Specifically, transcriptional regulation encompasses three major mechanisms: (1) transcription factor-mediated gene expression control, (2) histone modifications, and (3) DNA methylation. At the post-transcriptional level, regulation is primarily achieved through (1) non-coding RNAs, (2) RNA-binding proteins, and (3) RNA modifications. Post-translational regulation is predominantly mediated through diverse protein post-translational modifications. The transcriptional and post-transcriptional mechanisms primarily modulate the expression levels of serine metabolic enzymes, while post-translational modifications exert more diverse effects by altering the activity, protein stability or cellular localization of these enzymes. These regulations collectively modulate serine metabolism to influence tumor progression, offering promising targets for tumor-specific therapeutic interventions.

## Introduction

Metabolic reprogramming represents a pivotal hallmark of cancer, serving as a critical adaptive mechanism that not only fulfills the heightened bioenergetic and biosynthetic demands of malignant cells, but also modulates tumor microenvironment and resistance to chemotherapy [1]. While the Warburg effect (aerobic glycolysis) remains the prototypical example of oncogenic metabolic adaptation [2], accumulating evidence highlights the critical role of amino acid metabolic rewiring in tumor progression [3]. Particularly noteworthy is that serine metabolism has become a focal point in cancer research due to its pleiotropic regulatory functions, and has been found to be enhanced in multiple cancer types [4]. Serine metabolism encompasses two interconnected branches: *de novo* serine synthesis and catabolism. The serine synthesis pathway (SSP) converts the glycolytic intermediate 3-phosphoglycerate (3-PG) to serine through sequential catalysis by phosphoglycerate dehydrogenase (PHGDH), phosphoserine aminotransferase 1 (PSAT1), and phosphoserine phosphatase (PSPH) [5]. Conversely, serine catabolism is mediated by serine hydroxymethyltransferase (SHMT), which converts serine to glycine and a one-carbon unit [5]. SHMT exists as two distinct isoforms with compartment-specific localization: SHMT1 in the cytoplasm and SHMT2 in the mitochondria [6].

**Figure 1:**
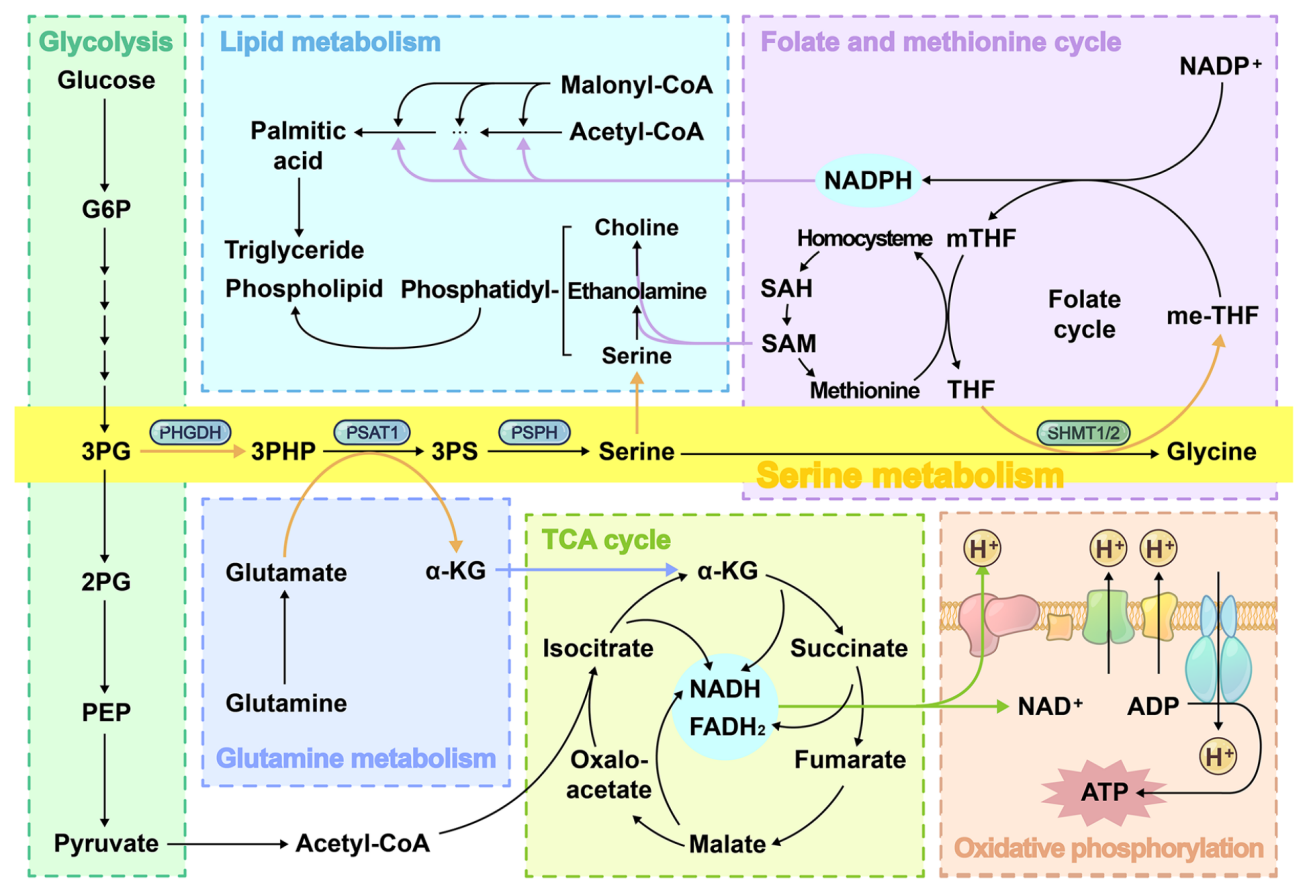
Figure 1 Serine metabolism and its crosstalk with other metabolic pathways Serine biosynthesis originates from a glycolytic intermediate, which is coupled with glutamine metabolism and contributes to energy production. Its catabolism generates glycine and one-carbon units, thereby driving the folate and methionine cycles to support lipid metabolism. 2-PG: 2-phosphoglycolate; 3-PHP: 3-phosphohydroxypyruvate; ADP: adenosine diphosphate; G6P: glucose-6-phosphate; mTHF: 5-methyltetrahydrofolate; PEP: phosphoenolpyruvate; SAH: S-adenosylhomocysteine; THF: tetrahydrofolate.

Serine metabolism plays a pivotal role in tumor metabolic reprogramming, acting as a central node that integrates with multiple metabolic pathways ( Figure 1) to support cancer cell proliferation, survival, and adaptation to stress [5]. One of its most prominent connections is with glycolysis. As a major glycolytic intermediate, 3-PG serves as the entry substrate into the SSP, thereby coupling serine biosynthesis directly to glucose catabolism. Serine, in turn, serves as an allosteric activator of pyruvate kinase M2 (PKM2) [7], enhancing glycolytic flux and promoting the Warburg effect. Another crucial amino acid metabolic reprogramming in tumors involves glutamine metabolism, which supplies α-ketoglutarate (α-KG) to the tricarboxylic acid (TCA) cycle. Cytosolic glutaminase converts glutamine to glutamate, while PSAT1 generates both 3-phosphoserine (3-PS) and α-KG through its transaminase activity. The PSAT1-derived α-KG replenishes the TCA cycle to provide reducing equivalents for oxidative phosphorylation (OXPHOS) and adenosine triphosphate (ATP) production, a critical energy source particularly under glutamine blockade conditions [8].

Serine catabolism directly interconnects with the folate cycle through SHMT-mediated generation of 5,10-methylenetetrahydrofolate (me-THF) during glycine synthesis, which enters the folate cycle to produce one-carbon units and nicotinamide adenine dinucleotide phosphate (NADPH) [9]. The resulting NADPH further supports OXPHOS and ATP production. The folate cycle also links with the methionine cycle to generate the universal methyl donor S-adenosylmethionine (SAM), thereby extending its functional repertoire [10]. In lipid metabolism, serine participates directly or indirectly (via folate and methionine cycles) in membrane lipid formation. It serves as the direct precursor for phosphatidylserine synthesis and, through methionine cycle-derived choline, contributes to phosphatidylcholine production. Additionally, NADPH generated through these pathways facilitates fatty acid synthesis by fueling multiple enzymatic steps [11].

Beyond its involvement in coordinating other metabolism pathways, serine synthesis establishes intricate connections with nutrition-sensitive signaling cascades, particularly forming a critical metabolic-signaling axis with mechanistic target of rapamycin complex 1 (mTORC1) to support tumor growth [12]. As a primary mTOR complex frequently dysregulated in human cancers, mTORC1 fundamentally promotes cell proliferation through regulating both protein synthesis and metabolic reprogramming [13]. The *de novo* serine synthesis pathway sustains mTORC1 activity to maintain proliferative capacity [14] while reciprocally, mTORC1 enhances serine metabolism by upregulating activating transcription factor 4 (ATF4) expression—the master transcriptional regulator of serine metabolic enzymes [ 15, 16] . Taken together, in the context of tumor metabolic reprogramming, serine metabolism serves as a metabolic hub, coordinating biosynthetic, bioenergetic, and signaling pathways. Its interactions with glycolysis, OXPHOS, the mTOR signaling, and other metabolic networks enable cancer cells to adapt to dynamic microenvironments, driving tumorigenesis and therapeutic resistance.

**Figure 2:**
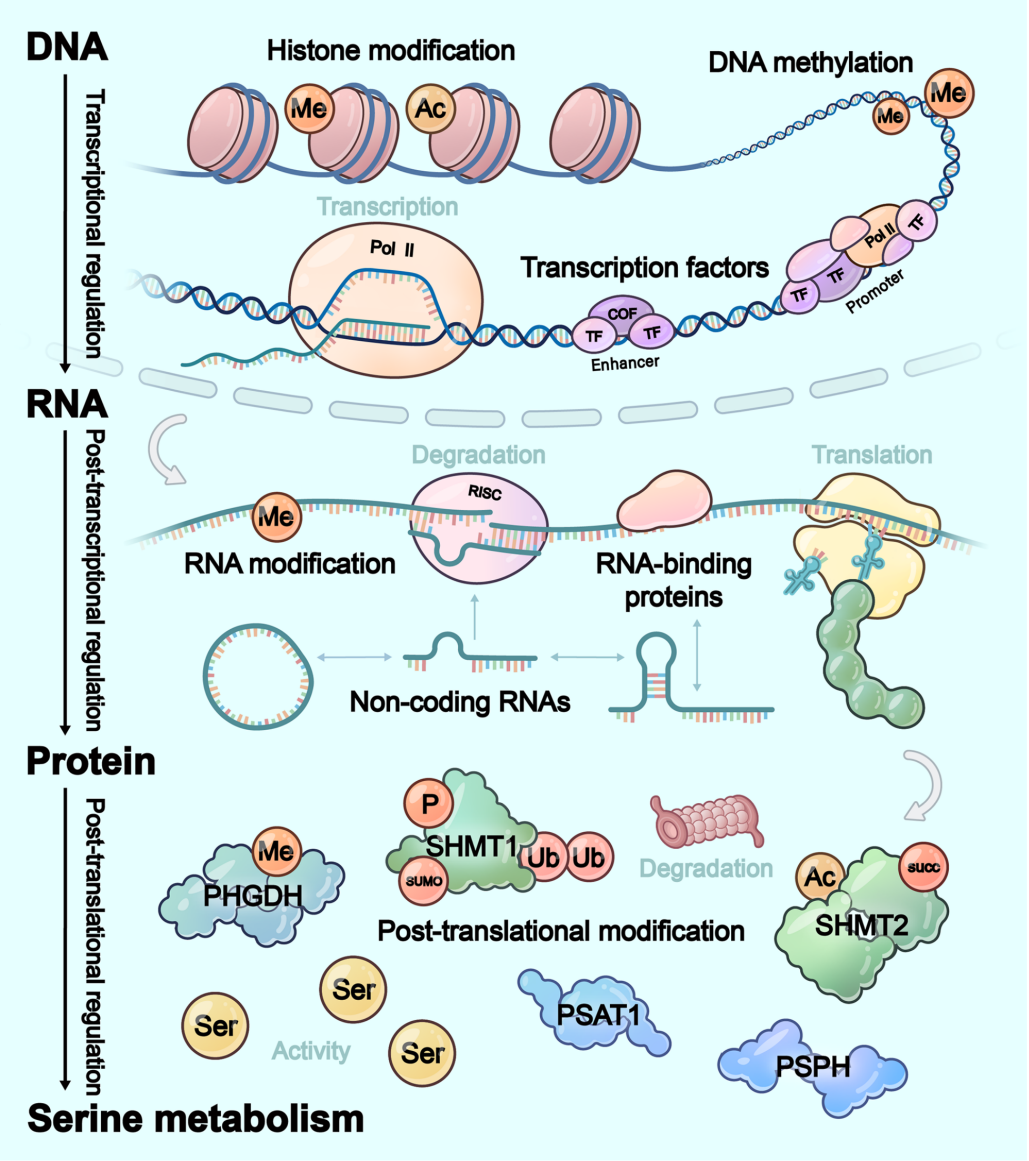
Figure 2 Regulation of the expression of serine metabolic enzymes The regulation of serine metabolic enzymes operates through transcriptional regulation (transcription factors and epigenetic modifications), post-transcriptional regulation (non-coding RNAs, RNA-binding proteins, and RNA modifications), as well as post-translational regulation (post-translational modifications). Ac: acetylation; COF: co-transcription factor; Me: methylation; P: phosphorylation; Pol II: RNA polymerase II; RISC: RNA-induced silencing complex; SUMO: SUMOylation; Ub: ubiquitination.

As a hallmark of metabolic reprogramming, serine metabolic enzymes are frequently upregulated across malignancies. Elevated expressions of most of these enzymes are observed in lung, breast, and colon cancers [ 17– 20] , with several specific enzymes overexpressed in gastric and liver cancers ( *e.g*., PSPH, SHMT2) [ 19, 20] . Additional examples include PHGDH in melanoma, PSAT1 in esophageal squamous cell carcinoma (ESCC), PSPH in cutaneous squamous cell carcinoma, and SHMT1 in ovarian carcinoma [ 21– 23] . The mechanisms driving this upregulation are multifaceted. Genomic amplification at amplified loci—such as chromosome 1p12 (PHGDH) in melanoma and breast cancer, or chromosome 12q14.1 (SHMT2) in lymphoma—represents one contributing factor [ 24– 27] . Beyond copy number alterations, diverse tumor-specific mechanisms enhance the expression, stability, or activity of these enzymes, often involving distinct regulatory mechanisms across tumor types and coexisting regulatory layers within an individual cancer. Despite their therapeutic relevance, systematic classification of these regulatory modes is still lacking. Here, we categorize serine metabolism regulation into three overarching domains: transcriptional regulation, post-transcriptional regulation, and post-translational modifications ( Figure 2). We hope that this framework could synthesize current knowledge on serine metabolic reprogramming and inspire novel targeting strategies for precision oncology.

## Role of Serine Metabolic Reprogramming in Tumor Cell Biology

**Figure 3:**
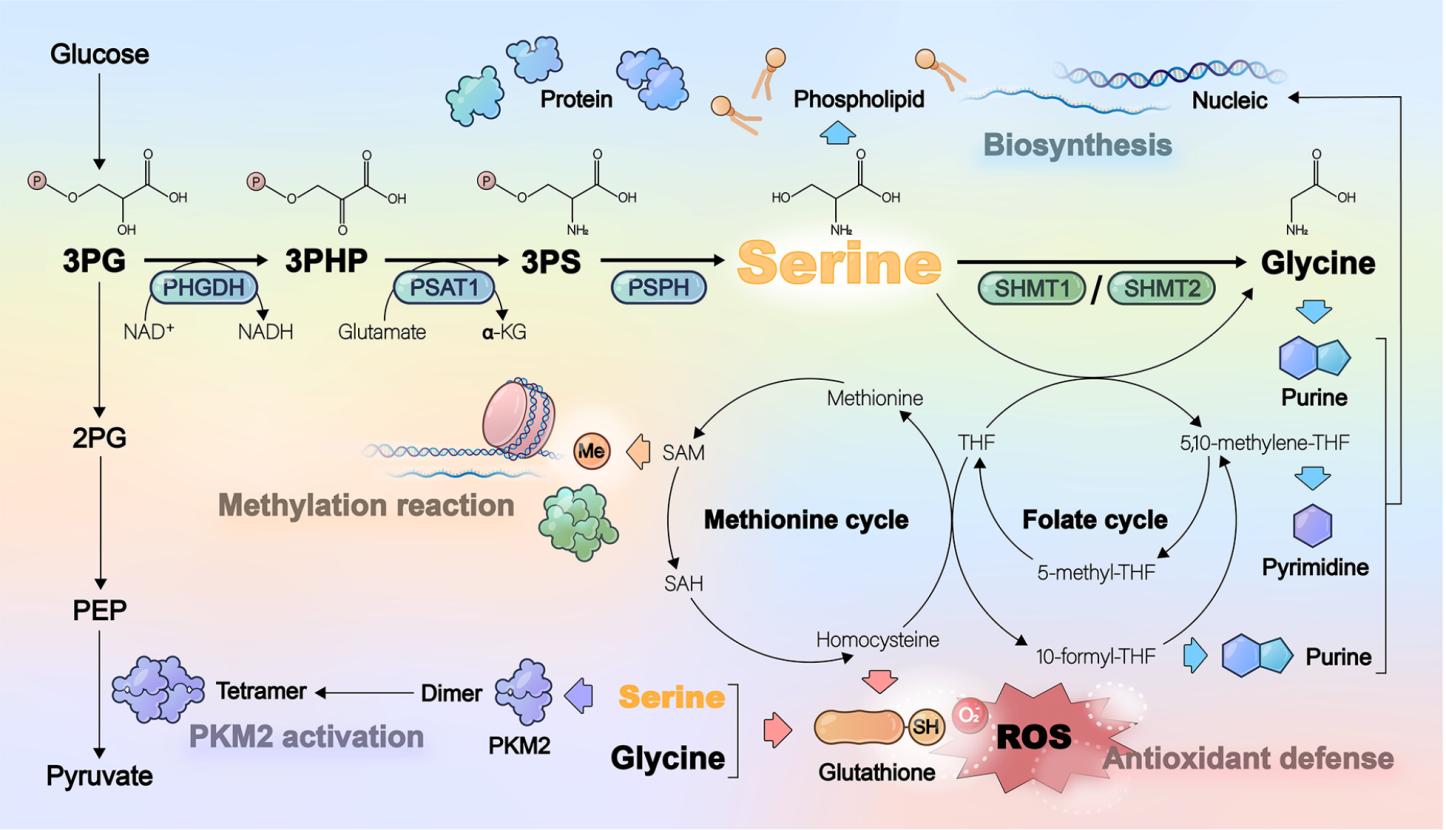
Figure 3 Serine metabolism and its important roles in cells Serine metabolism contributes to biosynthesis, redox homeostasis, and epigenetic regulation, playing pleiotropic roles in cellular growth and survival.

Although serine is a non-essential amino acid, its metabolic role in tumors cannot be overlooked ( Figure 3). Despite the Warburg effect enabling cancer cells to avidly uptake glucose and glutamine, these conventional carbon sources often fail to meet the biosynthetic demands of rapid proliferation [28]. Serine serves as a critical precursor for protein, nucleic acid, and lipid biosynthesis, partially compensating for this metabolic gap. Serine-derived glycine and the subsequent methionine cycle-generated methionine collectively facilitate protein translation and synthesis [29]. Glycine and one-carbon units further provide essential substrates for thymidine and purine nucleotide synthesis, directly fueling DNA and RNA production. In tumors, serine/glycine deprivation or PHGDH inhibition suppresses global protein and nucleotide synthesis, thereby impeding tumor growth *in vivo* [30]. Conversely, SHMT2-driven nucleotide biosynthesis promotes 5-Fluorouracil (5-FU) chemoresistance [31]. Additionally, serine contributes to membrane lipid homeostasis by phospholipid generation [32] and serves as the key substrate for *de novo* sphingolipid biosynthesis. Ceramide is a component of sphingolipids, synthesized from the condensation of serine and palmitoyl-CoA [33]. In breast cancer, serine restriction slows ceramide synthesis and causes mitochondrial fragmentation, thereby inhibiting proliferation [34]. Serine deficiency also leads to alanine substitution, resulting in deoxyceramide generation and subsequent toxic deoxysphingolipid accumulation, which ultimately induces mitochondrial dysfunction and suppresses colorectal tumor growth [33].

Serine metabolism exerts two additional crucial functions through redox homeostasis and methylation modifications. Glutathione (GSH) and NAD(P)H serve as essential reducing equivalents for maintaining cellular redox balance [35]. Serine metabolism supports GSH synthesis by providing glycine and driving the methionine cycle coupled with the transsulfuration pathway to generate cysteine [36]. Moreover, serine metabolism produces NADPH through oxidative reactions in the folate cycle, supplying reducing power to counteract oxidative stress and support biosynthesis in cancer cells [ 37, 38] . Under hypoxic conditions, SHMT2 activation regulates the NADP ^+^/NADPH ratio, and its knockdown elevates ROS levels to induce apoptosis [39]. Regarding methylation modifications, the methionine cycle interconnected with folate metabolism generates SAM, which functions as a methyl donor for DNA and histone methylation, thereby modulating epigenetic gene regulation [10]. This critical role is exemplified by PHGDH-mediated elevation of SAM levels that increased histone H3K4 trimethylation to upregulate cell adhesion genes and promote tumor cell migration [40], whereas SHMT2 downregulation reduces SAM levels, leading to decreased RNA N6-methyladenosine (m6A) methylation and consequent suppression of tumorigenesis [20].

Serine metabolic enzymes exhibit non-catalytic roles through protein-protein interactions beyond their catalytic functions in serine metabolism. For instance, PHGDH directly interacts with PKM2 and the oncogenic transcription factor FOXM1, enhancing their stability, while also engaging with eukaryotic translation initiation factor 4A1 (eIF4A1) and eIF4E to promote translation [ 41– 43] . Other serine metabolic enzymes, such as PSAT1 and SHMT2, have also been found to play similar roles. PSAT1 interacts with IQ motif-containing GTPase-activating protein 1 (IQGAP1) and NUMB, a protein numb homologue, thus activating downstream oncogenic pathways [ 44, 45] . Analogously, SHMT2 stabilizes β-catenin and hypoxia-inducing factor-1α (HIF-1α) through direct interactions [ 46, 47] . Through serine metabolism and these complex mechanisms, tumorigenesis is promoted at multiple stages by regulating cellular proliferation, stemness, and metastasis [48]. These findings highlight the diverse roles of serine metabolic enzymes in regulating key oncogenic pathways, further emphasizing their therapeutic potential in cancer treatment.

Given the critical role of serine metabolism in tumors, its inhibition holds significant therapeutic potential. Serine deprivation has shown promising antitumor effects in preclinical studies, effectively suppressing cancer cell proliferation. Dietary restriction of serine and glycine has been demonstrated to attenuate tumor growth in both xenograft and allograft models [49]. On the other hand, the *de novo* synthesis pathway as the main source of serine [50] renders its biosynthetic enzymes highly promising metabolic targets. Under physiological conditions where extracellular serine concentrations are insufficient to meet tumor metabolic demands, cancer cells activate endogenous serine synthesis to sustain proliferative capacity [51]. Furthermore, in hypoxic tumor cores where serine uptake is limited, cancer cells become dependent on endogenous synthesis [39]. Accumulating preclinical evidence demonstrates that genetic or pharmacological targeting of serine metabolic enzymes represents a promising strategy to inhibit tumor growth and metastasis [52]. For instance, *PHGDH* knockout or pharmacological inhibition suppressed tumor growth and metastasis, with enhanced efficacy observed under serine/glycine-deprived conditions in colon cancer xenografts [30]. Similarly, targeting PSAT1 may serve as a precision therapy for tumors harboring the p53-72Pro variant in xenograft models [53].

The blockade of serine catabolism has also demonstrated antitumor efficacy. In a T-cell acute lymphoblastic leukemia model using NSG mice, the SHMT1/2 inhibitor SHIN1 significantly reduced hCD45 ^+^ cells in bone marrow and spleen, effectively suppressing leukemia progression. This inhibitory effect was accompanied by impaired one-carbon metabolism and suppression of purine/pyrimidine synthesis, validating the *in vivo* antileukemic potential of targeting serine catabolism [54]. Similarly, SHMT2 inhibition—achieved via genetic silencing or small-molecule compounds (SHIN2)—effectively blocks Burkitt lymphoma progression *in vitro* and *in vivo* [55]. These findings collectively underscore the pivotal role of serine metabolism enzymes in cancer cell proliferation and highlight the significant clinical potential of targeting this metabolic pathway.

## The Regulation of Serine Metabolism

Reprogramming of serine metabolism in cancer is governed by a multi-tiered regulatory network, spanning transcriptional, post-transcriptional, and post-translational levels. These interconnected mechanisms collectively drive metabolic reprogramming, fueling tumor growth and therapy resistance, thereby revealing potential therapeutic targets for intervention.

### Transcriptional regulation

Transcriptional regulation, which occurs within the nucleus, involves the interplay between epigenetic and transcriptional control. Transcriptional regulation, primarily mediated by transcription factors, can modulate the transcription of serine metabolic enzymes through alterations in the expression levels. Epigenetic regulation, encompassing histone modifications and DNA methylation, contributes to the regulation of serine metabolic enzymes by influencing the access of transcription factors to their binding sites. Following this transcriptional layer of regulation, the mRNA levels of serine metabolic enzymes are expected to increase during cancer progression.

#### Histone modification

Histone modification is a heritable epigenetic modification, including histone methylation, acetylation, O-GlcNAcylation and lactylation, that affects gene transcription [ 56, 57] . EWS-FLI1 relies heavily on MLL1 (lysine methyltransferase 2A, KMT2A) and scaffold protein menin to maintain its tumorigenic properties in Ewing sarcoma [58]. Menin-MLL complex promoted MLL-dependent Histone H3 lysine 4 trimethylation (H3K4me3) on PHGDH gene promoter [59]. Inhibition of menin reduced the enrichment of H3K4me3 at the *PHGDH* promoter, resulting in reduced SSP gene expression and inhibition of tumorigenicity [60].

Methylation of H3K9 is one of the most well-studied histone modifications. H3K9 methyltransferase G9A, also known as EHMT2 (euchromatic histone lysine methyltransferase 2), activates the transcription of *PHGDH* and *PSAT1* by increasing the level of H3K9me1 in their promoter region and transcription start sites [ 61– 63] . Lysine-specific demethylase 4C (KDM4C), a histone lysine demethylase, also activates the transcription of serine metabolic genes in response to serine deprivation. Following KDM4C induction, its combination with the promoters of PHGDH and PSAT1 was significantly enhanced, thus decreasing H3K9me3 and increasing H3K9me1 levels of the above genes, leading to transcriptional activation.

Acetylation modifications similarly influence metabolic transcription. p300 and its paralog CREB-binding protein (CBP) mediate H3K18/H3K27 acetylation and promote PSPH/PSAT1 expression in HCC tissues, with inhibition of p300 demonstrating potent anti-tumor effects through promoter histone deacetylation *in vitro* and *in vivo* [64].

#### DNA methylation

DNA methylation, catalyzed by DNA methyltransferases (DNMTs), is a methyl modification on the fifth carbon of cytosine (5-methylcytosine) and usually occurs on symmetric CpG dinucleotides [ 65, 66] . Emerging evidence links DNA methylation patterns to serine metabolic reprogramming in malignancies. Global DNA hypomethylation in breast cancer correlates with elevated PHGDH expression [67], though the underlying regulatory mechanisms remain unclear.

Locus-specific methylation events demonstrate more defined regulatory roles. The Cg14476101 site within PHGDH’s first intron shows methylation levels inversely proportional to transcript abundance across tissues [ 24, 26, 67, 68] . In acute myeloid leukemia, DNA methyltransferase 3A (DNMT3A) mutations compromise methylation function and upregulate transcription of *PSPH*, *PSAT1*, and other genes involved in glutathione (GSH) synthesis [69]. Methylations of these genes are not common in cancer, and how these genes are methylated remains unknown.

#### Transcription factors

**Figure 4:**
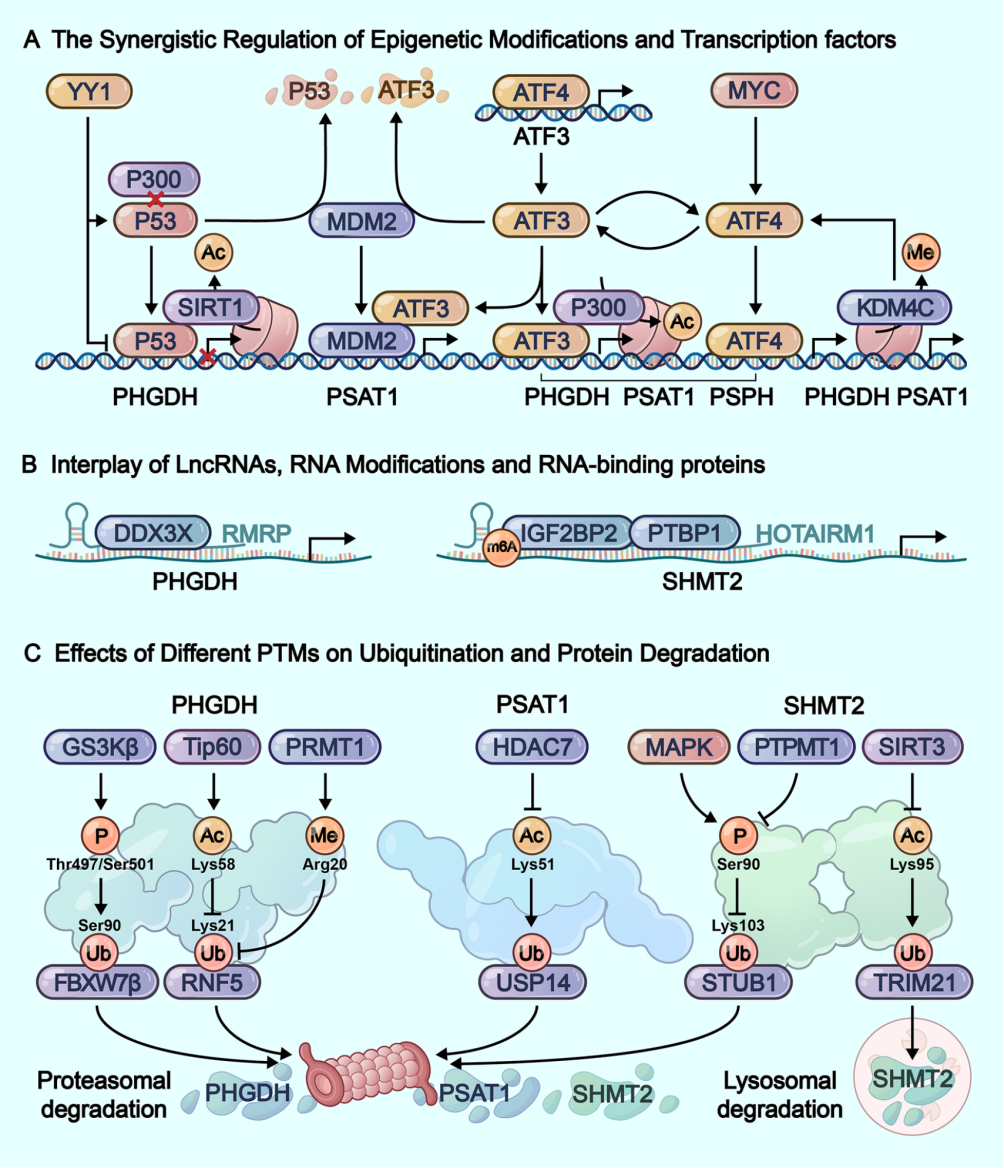
Figure 4 Within-layer regulatory interactions in serine metabolism (A) The transcriptional regulation of serine metabolic enzymes is orchestrated by a complex network of transcription factors and epigenetic modifiers that collectively fine-tune gene expression in a coordinated manner. (B) At the post-transcriptional level, serine metabolic enzyme expression is modulated through the interplay of lncRNAs, RNA-binding proteins, and RNA modifications, which collectively regulate mRNA stability and translation efficiency. (C) Crosstalk between various post-translational modifications orchestrates the ubiquitin-mediated degradation of key serine metabolic enzymes.

Transcription (TFs) factors are trans-acting factors that regulate the transcription of genes by binding to gene cis-acting elements in promoters or enhancers, either facilitating transcription initiation complex assembly [ 70, 71] or recruiting co-activators to form cis-regulatory modules [ 72– 74] . Key TFs modulating serine metabolic enzymes are summarized in Table 1. These transcription factors are not entirely independent, with their interrelationships illustrated in Figure 4A.

**Table:** **Table 1** The transcription factors of the enzymes in serine metabolism

TFs	Targets	Binding sites	Effect	Samples or DNA source	Ref.
ATF4	*PHGDH*	Promoter	+	Non-small cell lung cancer A549 cells (A549)	[ 75, 76]
*PSAT1*	Promoter	+	Breast cancer cells (BT-549 and MDA-468); periodontal ligament stem cells; non-small cell lung cancer A549 cells (A549)	[ 18, 76, 77]
*PSPH*	Unknown	+	HEK293T cells	[78]
*SHMT2*	Promoter	+	Non-small cell lung cancer A549 cells (A549)	[76]
ATF3	*PHGDH*	Enhancer, promoter	+	Colon cancer cells (HCT116); glioma cells (U87)	[ 79, 80]
*PSAT1*	Promoter	+	Colon cancer cells (HCT116)	[79]
*PSPH*	Intron 2	+	Colon cancer cells (HCT116)	[79]
MYC	*PHGDH*	The E-box close to the transcription start sites	+	Hepatocellular carcinoma cells (Hep3B)	[38]
*PSAT1*	The E-box close to the transcription start sites	+
*PSPH*	The E-box close to the transcription start sites	+
*SHMT1*	The E-box in promoter and conserved non-exonic regions	+	Primary human fibroblasts; human genomic library	[ 81, 82]
*SHMT2*	The E-box in promoter and conserved non-exonic regions	+
MYCN	*PHGDH*	Promoter	+	Neuroblastoma cells (BE(2)-C)	[83]
HIF-1	*PHGDH*	The HRE in promoter	+	Glioblastoma (U87MG)	[84]
*PSAT1*	The HRE in promoter
*PSPH*	The HRE in promoter
*SHMT2*	The HRE in promoter (under hypothesis)	+	Neuroblastoma cells (Kelly)	[39]
P53	*PHGDH*	Promoter	-	BLCA cell line (RT-4)	[85]
YY1	*PHGDH*	Promoter	+	BLCA cell line (RT-4)	[85]
YY2	*PHGDH*	Promoter	-	Colon cancer cells (HCT116)	[86]
MDM2	*PSAT1*	Promoter	+	Breast cancer cells (MDA-468)	[87]
ZEB1	*PHGDH*	Promoter	+	Hepatocellular carcinoma cells (MHCC-97H)	[88]
Sp1	*PHGDH*	Promoter	+	Human genomic library	[89]
NF-Y	*PHGDH*	Promoter	+	Human genomic library	[89]
c-Jun	*PSAT1*	Promoter	+	Liver cancer cells (Bel-7402 and SMMC-7721)	[90]
HOXD8	*SHMT1*	Promoter	+	HEK293T cell; renal cell carcinoma cells (ACHN)	[91]
WT1	*SHMT1*	Promoter	+	Ovarian cancer cells (PEO4)	[23]
ERRα	*SHMT2*	Promoter	+	Breast cancer cells (BT-474)	[92]
STAT3	*SHMT2*	Promoter	+	Prostate cancer cells (LNCaP and DU145)	[93]
TCF4	*SHMT2*	Open reading frame	+	Colorectal cancer cells (HCT116 and DLD-1)	[46]

Abbreviations: Sp1, specificity protein 1; WT1: Wilms tumor 1.

##### ATF4 and ATF3

Activating transcription factor 4 (ATF4), a key member of the cAMP-responsive element-binding protein family, functions as a stress-responsive transcription factor frequently overexpressed in malignancies [ 94– 96] . It orchestrates diverse cellular processes, including amino acid/lipid metabolism, endoplasmic reticulum stress, autophagy, energy homeostasis, and inflammatory responses [ 96– 98] . Notably, ATF4 directly binds to promoter regions to transcriptionally upregulate serine biosynthesis enzymes PHGDH, PSAT1, PSPH, and SHMT2 [ 18, 75– 77, 99] . This regulatory mechanism was found to promote tumor progression in breast cancer, non-small cell lung cancer (NSCLC), hepatocellular carcinoma (HCC), and Burkitt lymphoma [ 63, 76, 78, 99] . Particularly, in ER-negative breast cancer models, ATF4-mediated PSAT1 upregulation drives tumor proliferation via the glycogen synthase kinase 3β (GSK3β)/β-catenin/cyclin D1 signaling axis [26].

ATF3, a stress-responsive member of the ATF4 family, can also coordinate transcriptional programs under cellular stress [ 96, 100] . This transcription factor is both transcriptionally regulated by ATF4 and functionally involved in serine metabolism regulation during nutrient restriction. Mechanistic studies reveal that ATF3 binds to promoter regions of *PHGDH*, *PSAT1*, and *PSPH*, recruiting the p300 histone acetyltransferase to enhance gene expression, thereby sustaining colon cancer cell proliferation under serine-deprived conditions [79]. Furthermore, ATF4-ATF3 protein interaction stabilizes ATF4 through complex formation, establishing a feedforward loop that amplifies serine metabolic enzyme expression [30].

##### MYC family

The MYC oncogene family, including MYC (also known as c-Myc) and its paralogs MYCL and MYCN, controls diverse cellular processes, from cell cycle progression to metabolic reprogramming [ 101– 103] . Oncogenic activation of MYC family members occurs in most human malignancies, driving tumorigenesis through genome-wide transcriptional regulation [ 103, 104] . Notably, MYC proteins coordinate transcriptional activation of serine metabolic enzymes through both direct and indirect mechanisms.

MYC directly binds to the E-box sequences near the transcription start sites of *PHGDH*, *PSAT1*, and *PSPH*, transcriptionally upregulating these enzymes to stimulate SSP flux. This metabolic rewiring enhances glutathione biosynthesis and nucleotide production, sustaining tumor cell proliferation under nutrient stress [38]. Furthermore, the MYC regulatory network could extend to another two targets, *SHMT1* and *SHMT2* [81]. MYC directly binds to their promoters, which is consistent with the MYC-dependent expression of these genes in rat fibroblasts [82]. Additionally, evidence suggested that MYC indirectly regulates the transcription of *PHGDH* and *PSAT* partly by regulating ATF4 [99]. Another study demonstrated that MYC can activate the upstream pathway of ATF4, which may account for the underlying mechanism [105]. Notably, MYCN demonstrates similar metabolic governance. MYCN binds to two putative regulatory elements in the *PHGDH* promoter, with MYCN-amplified neuroblastoma exhibiting elevated serine biosynthesis and PHGDH-dependent growth [83]. Additionally, the expression of SHMT2 is also upregulated in MYCN-amplified neuroblastoma cells [39], suggesting that MYCN may also regulate the transcription of *SHMT2*.

##### HIF-1

HIF-1 heterodimer comprises oxygen-sensitive HIF-1α and constitutively expressed HIF-1β subunits. Under hypoxia, stabilized HIF-1α accumulates and translocates to the nucleus, where HIF-1α heterodimerizes with HIF-1β and binds to the hypoxic response elements (HREs) in target gene promoters to drive cancer adaptation [106]. In glioblastoma, HIF-1 directly activates *PHGDH*, *PSAT1*, and *PSPH* transcription through HRE-containing promoter interactions [84]. Notably, serine/glycine deprivation induces AMP-activated protein kinase (AMPK)-dependent HIF-1α activation, subsequently upregulating SSP genes [84]. Furthermore, HIF-1 has also been hypothesized to mediate *SHMT2* transcription by binding to the dual HRE on its promoter, establishing a stress response mechanism under hypoxia [39].

##### p53, MDM2, and YY

The tumor suppressor p53, along with its associated proteins MDM2 (mouse double minute 2 protein, or HDM2 in humans) and YY (Yin Yang), are also involved in the regulation of serine metabolic enzymes. p53 coordinates stress signaling through complex interactions with transcriptional regulators [107]. MDM2, a negative regulator of p53, acts as an E3 ubiquitin ligase and is capable of mediating p53 ubiquitination and proteasome degradation [108]. Notably, MDM2 also exhibits p53-independent oncogenic functions, expanding its role in tumorigenesis [87]. The zinc finger protein YY1 functions as a negative p53 modulator by binding to MDM2 and enhancing the ubiquitination of p53, while YY2 is the retrotransposon-derived paralogue of YY1 [ 108– 111] .

Wild-type p53 suppresses *PHGDH* transcription through recruiting sirtuin 1 (SIRT1) to its promoter, leading to the reduction of histone H3 acetylation (H3Kac) [85]. This tumor-suppressive mechanism induces apoptosis in serine-deprived melanoma by limiting PHGDH expression [112]. The p53-PHGDH axis is competitively regulated by YY1, which shares overlapping DNA-binding motifs with p53 at the *PHGDH* promoter [85]. YY1 further amplifies its oncogenic effects by disrupting p53-p300 interactions while enhancing p53-MDM2 binding, effectively downregulating p53 activity [111]. In p53-mutant contexts, YY1-driven PHGDH overexpression promotes bladder tumorigenesis, whereas YY2 shows opposing function through directly repressing *PHGDH* transcription [ 85, 86] . MDM2 exhibits dual regulatory roles in this network. Beyond its canonical p53 degradation function, MDM2 paradoxically binds to the promoter regions of *PHGDH*, *PSAT1*, and *PSPH* to enhance their transcription [87].

Interestingly, MDM2 engages in regulatory interaction with ATF3/4, wherein they mutually facilitates the transcriptional programs, including PSAT1 [87]. Furthermore, MDM2 and ATF3 establish a regulatory loop: MDM2 modulates the ubiquitination-mediated degradation of ATF3, while ATF3 suppresses the transcription of p53 target genes, including *MDM2* [ 113, 114] . Collectively, these findings reveal an interconnected regulatory axis where p53, YY proteins, MDM2, and ATF3/4 converge to orchestrate serine metabolic plasticity.

##### Additional transcriptional factors

Beyond the core regulatory network, multiple factors modulate serine metabolic genes. Zinc Finger E-Box Binding Homeobox 1 (ZEB1) activates *PHGDH* transcription by binding to non-classical binding sites, enhancing SSP flux to drive HCC invasion, ROS resistance, and sorafenib tolerance [88]. TCF4, as an effector of Wnt/β-catenin signaling, could bind to the *SHMT2* open reading frame and increase the transcription of *SHMT2*. SHMT2 also interacts with β-catenin in the cytoplasm and inhibits β-catenin degradation mediated by ubiquitination, which forms a SHMT2/β-catenin positive feedback loop and promotes the progression of CRC [46]. Multiple transcription factors regulating serine metabolism, such as EWS/FLI1, homeobox D8 (HOXD8), and signal transducer and activator of transcription 3 (STAT3), have been identified to bind directly to the promoters of these enzyme genes [ 23, 90, 93] . Given the functional overlap with aforementioned transcriptional regulators, their mechanistic details are not discussed separately but are comprehensively summarized in Table 1.

#### The synergistic regulation of epigenetic modifications and transcription factors

The cooperative regulation between epigenetic modifications and transcription factors is manifested in their collaborative control over metabolic enzyme gene transcription ( Figure 4A). For instance, ATF3 interacts with the histone acetyltransferase p300 to activate the transcription of serine metabolic enzyme genes, whereas p53 recruits the histone deacetylase SIRT1 to specifically reduce PHGDH histone acetylation levels and suppress its transcription [ 64, 85] . Although p300/CBP-associated factor (PCAF) has been identified as an interacting partner of ATF4 under amino acid starvation, their functional cooperation in serine metabolism remains unexplored [115]. On the other hand, transcription factors regulating serine metabolic enzymes are themselves subject to epigenetic modulation. For example, ATF4 is a direct target of KDM4C, and their interaction cooperatively enhances the transcription of serine metabolic genes [116].

### Post-transcriptional regulation

Following transcriptional regulation, tumors exhibit increased basal mRNA expression levels of serine metabolic enzyme genes. However, during the transition from mRNA synthesis to translation, multiple regulatory factors modulate mRNA abundance through post-transcriptional regulation. This cytoplasmic process is mediated primarily by three key components: non-coding RNAs, RNA-binding proteins, and RNA modifications. These elements exert direct control over mRNA molecules, thereby establishing RNA-level regulation. Through their coordinated interactions, these mechanisms ultimately enhance both mRNA stability and translational efficiency.

#### Non-coding RNAs

Non-coding RNAs (ncRNAs) constitute ~90% of human transcripts and play a regulatory role in various life processes without protein-coding [ 117, 118] . Complementing transcriptional regulation, the three principal ncRNA classes, including microRNAs (miRNAs), long noncoding RNAs (lncRNAs), and circular RNAs (circRNAs), demonstrate significant regulatory capacity over serine metabolic enzymes, establishing multilayered control of cancer-associated metabolic reprogramming.

##### miRNAs

miRNAs, ~22-nucleotide noncoding RNAs, regulate serine metabolism through sequence-specific interactions with directly targeted mRNAs [119]. The miRNA-loaded Argonaute (AGO) protein combines with other cofactors to form miRISC (miRNA-induced silencing complex), which induces mRNA decay and translation inhibition by binding to the complementary sequences in the 3′-untranslated regions (3′UTRs) of the target mRNA [ 120– 122] . Numerous miRNAs targeting serine metabolic enzymes exhibit decreased expression in malignancies, with similar mechanisms. Given that the mechanisms underlying the downregulation of most miRNAs remain unknown, this represents a valuable research direction. Here, we introduce several miRNAs with broad impact or well-characterized functions. All relevant miRNAs can be found in Table 2.

**Table:** **Table 2** The microRNAs that regulate the expressions of serine metabolic enzymes

MicroRNAs	Targets	Expression	Samples	Ref.
miR-34b-5p	*PHGDH*	↓	Breast cancer (T47D cells)	[123]
miR-876-5p	*PHGDH*	↓	Breast cancer (T47D cells)	[123]
miR-940	*PHGDH*	↓	Colorectal cancer (HCT8 and DLD1 cells)	[124]
miR-107	*PHGDH*	Unknown	Hepatocellular carcinoma (PLC cells)	[125]
miR-103a-3p	*PHGDH*	Unknown	Hepatocellular carcinoma (PLC cells)	[125]
miR-195-5p	*PSAT1*	↓	Breast cancer (BT-549 cells)	[126]
miR-145-5p	*PSAT1*	↓	Breast cancer (LCC9, ZR-751, and ZR-75–1-4-OHT cells)	[123]
↓	Colon cancer (HT29 cells)	[127]
↓	Ovarian cancer (SKOV3 and OV90 cells)	[128]
miR-424-5p	*PSAT1*	↓	Breast cancer (LCC9, ZR-751, and ZR-75–1-4-OHT cells)	[123]
miR-424	*PSAT1*	↓	Colorectal cancer (HCT116 and RKO cells)	[129]
miR-365	*PSAT1*	↓	Esophageal squamous cell carcinoma (EC109 and EC9706 cells)	[130]
miR-340	*PSAT1*	↓	Esophageal squamous cell carcinoma (EC109 and EC9706 cells)	[131]
miR-497-5p	*PSAT1*	↓	Gastric cancer (SGC-7901 cells)	[132]
miR-5195-3p	*PSAT1*	↓	Non-small cell lung carcinoma (A549 and H1299 cells)	[133]
miR-15a-5p	*PSAT1*	↓	Non-small cell lung carcinoma (A549 cells)	[134]
miR-15b-5p	*PSAT1*	↓	Non-small cell lung carcinoma (A549 cells)	[134]
miR-218-5p	*SHMT1*	↑	NK cells from lung adenocarcinoma patients	[135]
miR-198	*SHMT1*	↓	Lung adenocarcinoma (A549 and Calu-3 cells)	[136]
miR-615-5p	*SHMT2*	↑	Hepatocellular carcinoma (HepG2, Hep3B, Huh1, Huh7, and HLE cells)	[137]
↓	Non-small cell lung cancer (A549 cells)	[138]
miR-193b	*SHMT2*	Unknown	Breast cancer (MCF-7 cells)	[139]
miR-370	*SHMT2*	↓	Osteoarthritis chondrocytes	[140]
miR-149-5p	*SHMT2*	Unknown	Breast cancer (SKBR3 and BT-549 cells)	[141]
miR-449a	*SHMT2*	↓	Gastric cancer (AGS and HGC27 cells)	[142]

Among the five serine metabolic enzymes, PSAT1-targeting miRNAs have been most extensively studied. Notably, miR-195-5p shows consistent downregulation and functional activity across multiple cancer types, mediating its pan-cancer effects through direct PSAT1 regulation [143]. In triple-negative breast cancer (TNBC), rescue experiments confirmed miR-195-5p-mediated tumor suppression via direct PSAT1 regulation, as PSAT1 overexpression reversed these effects both *in vitro* and *in vivo* [126]. Similarly, in estrogen receptor α-positive (ER+) breast cancer, miR-195-5p downregulation led to PSAT1 elevation through 3′UTR binding [123], with parallel regulatory mechanisms observed in ovarian cancer [128]. miR-34b-5p is also a tumor-suppressive miRNA due to its cooperation with p53 [144]. In ER ^+^ breast cancer, miR-34b-5p directly targetes the 3′UTR of *PHGDH* mRNA, suppressing its expression and inhibiting tumor progression [123]. miR-193b coordinates tumor suppression by simultaneously regulating enzymes such as SHMT2 and YWHAZ (14-3-3ζ), while also inhibiting estrogen receptor-α (ERα), thereby dual-targeting steroid-dependent growth pathways [139].

##### lncRNA and circRNA sponge effects on miRNAs

miRNAs are commonly regulated via competitive interactions with lncRNAs and circRNAs. lncRNAs (RNA molecules exceeding 200 nt in length) constitute a significant proportion of the transcriptome [145]. These noncoding transcripts exhibit remarkable functional diversity through their DNA, RNA, and protein interactions [ 146– 148] , thereby regulating a wide range of biological processes, such as cell cycle, gene expression, immune response, and cell differentiation [ 149– 152] . CircRNAs are single-stranded covalently closed transcripts lacking 5′ caps and 3′ poly(A) tails [ 153, 154] . Generated through back-splicing of precursor mRNAs, circRNAs modulate tumor progression through transcriptional regulation, miRNA sponging, and protein sequestration [ 155– 158] . In serine metabolism, both lncRNAs and circRNAs can act as miRNA sponges, also called competitive endogenous RNA (ceRNA), complementing miRNA-mediated regulation ( Tables 3 and 4).

**Table:** **Table 3** The lncRNAs that regulate the expressions of serine metabolic enzymes

lncRNAs	Targets	Expression	Mechanism	Samples	Ref.
linc01564	*PHGDH*	↑	Sponge miR-107 and miR-103a-3p	Hepatocellular carcinoma (PLC cells)	[125]
RMRP	*PHGDH*	↑	Enrich DDX3X on 3′UTR of *PHGDH* mRNA	Ovarian cancer (SKOV3 and A2780 cells)	[159]
PlncRNA-1	*PHGDH*	↓	Unknown	Breast cancer (MCF-7 and MDA-MB-468 cells)	[160]
WT1-AS	*PHGDH* *PSPH*	↓	Unknown	Gastric cancer stem cells	[161]
MEG8	*PSAT1*	↑	Sponge miR-15a-5p and miR-15b-5p	Non-small cell lung carcinoma (A549 cells)	[134]
MEG3	*PSAT1*	↓	Unknown	Esophageal squamous cell carcinoma (EC109 cells)	[162]
CASC9	*PSAT1*	Unknown	May sponge many targeted miRNAs	Lung adenocarcinoma	[163]
RP4-694A7.2	*PSAT1*	↑	Unknown	Hepatocellular carcinoma (SK-Hep-1 cells)	[164]
Gm15290	*SHMT2*	↑	Sponge miR-615-5p	Non-small cell lung carcinoma (A549 cells)	[138]
HOTAIRM1	*SHMT2*	↑	Enrich IGF2BP2 and PTBP1 on *SHMT2* mRNA	Glioblastoma (A172 cells)	[165]

**Table:** **Table 4** The circRNAs that regulate the expressions of serine metabolic enzymes

circRNAs	Targets	Expression	Mechanism	Samples	Ref.
circ_0062682	*PHGDH*	↑	Sponge miR-940	Colorectal cancer (HCT8 and DLD1 cells)	[124]
circ_0015756	*PSAT1*	↑	Sponge miR-145-5p	Ovarian cancer (SKOV3 and OV90 cells)	[128]
circERBB2IP	*PSAT1*	↑	Sponge miR-5195-3p	Non-small cell lung carcinoma (A549 and H1299 cells)	[133]
hsa_circ_0025036	*SHMT1*	↑	Sponge miR-198	Lung adenocarcinoma (A549 and Calu-3 cells)	[136]
circ_0072995	*SHMT2*	↑	Sponge miR-149-5p	Breast cancer (SKBR3 and BT-549 cells)	[141]
circ_0063526	*SHMT2*	↑	Sponge miR-449a	Gastric cancer (AGS and HGC27 cells)	[142]

Owing to the consistent and well-defined mechanisms, we have elected to present a limited number of examples. In NSCLC, lncRNA MEG8 (maternally expressed genes 8) functions as a ceRNA by sequestering miR-15a-5p and miR-15b-5p, thereby counteracting their suppressive effects on PSAT1. This MEG8/miR-15a/b-5p/PSAT1 axis drove tumor progression through enhanced serine metabolic flux in both preclinical models and clinical specimens [134]. circ_0062682 is upregulated in CRC and correlates with poor prognosis as a miR-940 sponge. Genetic silencing of circ_0062682 suppressed CRC proliferation via PHGDH downregulation, highlighting its therapeutic potential through the miR-940/PHGDH axis [124]. It should be noted that the regulation of serine metabolism by lncRNAs involves additional complexity, such as interactions with RNA-binding proteins (RBPs), which will be elaborated upon later in this section.

#### RNA-binding proteins

RNA-binding proteins (RBPs) orchestrate mRNA processing, stability, splicing, and translation through direct RNA interactions, critically regulating post-transcriptional gene expression [ 166, 167] . In serine metabolism, RBPs primarily enhance the expressions of metabolic enzymes via transcript stabilization or translational potentiation.

Epithelial splicing regulatory protein 1 (ESRP1) stabilizes *PHGDH* mRNA by binding to its 5′UTR in tamoxifen-resistant ER ^+^ breast cancer, linking RNA stability to therapy resistance [ 168, 169] . This reveals the metabolic regulatory function of ESRP1 beyond its canonical role in EMT (epithelial-to-mesenchymal transition)-related splicing [170]. Another RBP, fragile X-related protein-1 (FXR1), was also found to support the stability and translation of *PSAT1* mRNA. Isocitrate dehydrogenase 1 (IDH1) could reduce the expression of PSAT1 by blocking FXR1 from binding to its E3 ubiquitin ligase parkin [171]. In addition to these RBPs, numerous others, including m ^6^A writers and readers, are involved in RNA modification and will be introduced and discussed in the following subsections.

Intriguingly, the metabolic enzyme SHMT1 exhibits non-canonical RNA-binding activity, directly interacting with the 5′UTR (UTR2) of *SHMT2* mRNA to control its expression while suppressing its own enzymatic activity [172]. This dual functionality revealed bidirectional crosstalk between metabolic enzymes and RNA regulation.

#### RNA modification

Emerging evidence highlights RNA modifications as pivotal mechanisms in post-transcriptional control, with over 100 chemically distinct RNA modifications identified to date [173]. Among these, N6-methyladenosine (m ^6^A), the most abundant internal mRNA modification, regulates RNA splicing, translation, and stability through adenosine methylation at the nitrogen-6 position [173]. In serine metabolism, m ^6^A exerts partial regulatory effects through RNA-binding protein-mediated deposition and recognition.

In breast cancer, m ^6^A methylations were found in the exons and UTRs of the mRNAs of *PHGDH*, *PSAT1*, *PSPH*, and *SHMT2*. The m ^6^A writer RBM15 binds to RNA methyltransferase METTL3/14 (methyltransferase-like 3/14) and forms the m ^6^A writer complex, which mediates the m ^6^A methylation and enhances the stability of *PSAT1*, *PSPH* and *SHMT2* mRNAs [174]. Compared with *PSAT1*, *PSPH* and *SHMT2* mRNAs, the mRNA of *PHGDH* is less m ^6^A methylated. RBM15 does not mediate the methylation of *PHGDH* mRNA, but could upregulate PHGDH expression in a manner independent of methyltransferase activity [174]. HCC exhibits METTL3-mediated m ^6^A modification of *PHGDH*, *PSAT1*, and *PSPH* transcripts, with IGF2BP3 (insulin-like growth factor 2 mRNA binding protein 3) acting as the m ^6^A reader to stabilize these mRNAs [175]. The m ^6^A modification of *SHMT2* mRNA exons was found in glioblastoma. Similarly to IGF2BP3, IGF2BP2 could bind to the m ^6^A sites, promoting the stabilization and accumulation of *SHMT2* mRNA [176]. In CRC, METTL3-driven m ^6^A modification enhances PHGDH translation via eIF3i binding [159]. Collectively, these findings underscore that m ^6^A methylation not only augments the stability of serine metabolic enzyme mRNAs but also promotes their translation.

#### Interplay of lncRNAs, RNA modifications and RBPs

Complementing miRNA-mediated regulation by ceRNA networks, lncRNAs also coordinate serine metabolism through indirect mechanisms involving RBP interactions ( Table 3 and Figure 4B). For instance, lncRNA RMRP (RNA component of mitochondrial RNA processing endoribonuclease) is upregulated in platinum-resistant ovarian cancer and was demonstrated to have oncogenic potential by recruiting RNA-binding protein DDX3X (DEAD-box helicase 3 X-linked) to the 3′UTR of *PHGDH* mRNA, thereby enhancing PHGDH translation [176]. In glioblastoma, the lncRNA HOTAIRM1 stabilized *SHMT2* mRNA by scaffolding RNA-binding proteins IGF2BP2 and PTBP1 (polypyrimidine tract-binding protein 1). HOTAIRM1 facilitates their combination and their binding to *SHMT2* mRNA [165]. IGF2BP2, as an m ^6^A reader, mainly combines the coding sequence (CDS) of *SHMT2* mRNA, while PTBP1 could combine IGF2BP2 and *SHMT2* mRNA (CDS and 3′UTR), which maintains the expression of SHMT2 by inhibiting mRNA decay [165].

### Post-translational regulation

Transcriptional and post-transcriptional regulation collectively modulate RNA abundance of serine metabolic enzymes, yet remain insufficient for protein-level regulation. Tissue-wide analyses revealed that more than half of gene products exhibit discordant RNA-protein enrichment patterns [177], emphasizing the critical role of post-translational regulation. Notably, post-translational modifications constitute the predominant mechanism of such protein-level regulation.

#### Post-translational modification

Post-translational modification (PTM) is a biochemical process of the modification of proteins after translation and plays a key role in the regulation of protein activity, involving almost every aspect of cell structure and function [ 178, 179] . Major PTM types include phosphorylation, ubiquitination, SUMOylation (small ubiquitin-like modifier mediated modification), acetylation, methylation, glycosylation, and lipidation. Among them, phosphorylation, as one of the most extensively studied PTMs, affects ~30% of proteins and participates in almost all life processes of cells [180]. Ubiquitination is another widely existing modification that is often associated with protein activity or stability [181]. Different types of modifications affect protein function by altering the protein charge state, conformation, hydrophobicity, and stability [182]. The PTM landscape of serine metabolic enzymes is predominantly regulated through altered expressions of specific modifying enzymes. Mounting evidence underscores PTMs as pivotal regulatory mechanisms for serine metabolism enzymes ( Table 5).

**Table:** **Table 5** The modifications that regulate the expressions of serine metabolic enzymes

Enzymes	Sites	PTMs	Modifying/demodifying enzymes	Role of modification	Samples	Ref.
PHGDH	Arg20	Me	PRMT1	Inhibit the ubiquitination of K21 and increase the stability of PHGDH	Triple-negative breast cancer	[183]
Lys21	Ub	RNF5	Mediate the degradation of PHGDH by the proteasome pathway	Breast cancer	[ 183, 184]
Arg54	Me	PRMT1	Maintain the activity of PHGDH for substrate affinity	Triple-negative breast cancer	[183]
Ser55	P	PKCζ	Inhibit the enzymatic activity of PHGDH	Intestinal cancer	[185]
AMPK	Change the activity of PHGDH to catalyze malate to oxaloacetate and reduce nucleus NAD ^+^ levels	Pancreatic cancer	[186]
Thr57	P	PKCζ	Inhibit the enzymatic activity of PHGDH	Intestinal cancer	[185]
Lys58	Ac	Tip60; SIRT2	Inhibit the ubiquitination and degradation of PHGDH by disrupting the interaction with E3 ubiquitin ligase RNF5	Breast cancer	[184]
Thr78	P	PKCζ	Inhibit the enzymatic activity of PHGDH	Intestinal cancer	[185]
Lys146	Ub	Cul4A-DDB1-DCAF16 complex	Enhance the activity of PHGDH by promoting the tetramer formation and recruiting DnaJ	Colorectal cancer	[40]
Arg236	Me	PRMT1	Elevate activity of PHGDH by increasing substrate affinity	Hepatocellular carcinoma	[17]
Lys310	Ub	UCHL3	Mediate the degradation of PHGDH by the proteasome pathway	Colorectal cancer	[175]
Lys330	Ub	Parkin	Mediate the degradation of PHGDH by the proteasome pathway	Breast cancer and lung cancer	[187]
Ser371	P	p38	Promote PHGDH transport to nucleus	Pancreatic cancer	[186]
Thr497	P	GSK3β	Mediate the ubiquitination and degradation of PHGDH by FBXW7β	Colorectal cancer	[188]
Ser501	P	GSK3β	Mediate the ubiquitination and degradation of PHGDH by FBXW7β
–	Ub	FBXW7β	Mediate the degradation of PHGDH by the proteasome pathway
–	Ub	SYVN1	Mediate the degradation of PHGDH by the proteasome pathway	Bladder cancer	[189]
–	Ub	ASS1	Mediate the degradation of PHGDH by the proteasome pathway	Triple-negative breast cancer	[190]
–	Ub	Josephin-2	Mediate the degradation of PHGDH	Hepatocellular carcinoma; lung adenocarcinoma	[ 191, 192]
PSAT1	Lys51	Ac	HDAC7	Inhibit the K63-linked ubiquitination at this site (Lys51); promote the interaction with E3 ubiquitin ligase UBE4B	Lung adenocarcinoma	[193]
Ub	–	Enhance the interaction with USP14
–	Ub	UBE4B; USP14	Mediate the degradation of PSAT1 via the K48-ubiquitin-proteasome pathway
SHMT1	Lys38/ Lys39	Ub	ubc13	Promote SHMT1 nuclear export and increase its nuclear stability	Cervical cancer	[194]
SUMO	ubc9	Promote SHMT1 nuclear degradation
SHMT2	Ser90	P	MAPK1; PTPMT1	Maintain the stability of SHMT2 by inhibiting the ubiquitination by STUB1	Lung adenocarcinoma	[20]
	Lys103	Ub	STUB1	Mediate the degradation of SHMT2 via the K48-ubiquitin-proteasome pathway
SHMT2	Lys95	Ac	SIRT3	Inhibit the activity and stability of SHMT2 by destroying the tetramer structure and promoting the ubiquitination by TRIM21	Colorectal cancer	[195]
	–	Ub	TRIM21	Mediate the degradation of SHMT2 via the K63-ubiquitin-lysosome pathway
	Lys280	succ	SIRT5	Inhibit the activity of SHMT2 by preventing PLP binding and tetramer formation	Osteosarcoma	[196]

##### Ubiquitination and SUMOylation

Ubiquitination governs PHGDH homeostasis through E3 ligase-deubiquitinase pairs with divergent degradation mechanisms. Proteasomal degradation is mediated by multiple E3 ligases: Parkin (targeting Lys330) [187], RNF5 (modifying Lys21) [ 184, 183] , and synoviolin (SYVN1)/argininosuccinate synthase (ASS1) (sites uncharacterized) [189, 190]. Stabilizing ubiquitination occurs via the cullin 4A-based E3 ligase complex (Cul4A-DDB1-DCAF16 complex), which monoubiquitinates Lys146 to enhance PHGDH tetramerization and activity through DnaJ homolog subfamily A member 1 (DNAJA1) recruitment, driving SAM/H3K4me3 oncogenic signaling [40]. Deubiquitinases counterbalance proteasomal degradation and stabilize PHGDH: eIF3f disrupts FBXW7β (F-Box and WD Repeat Domain Containing 7 β)-mediated ubiquitination of PHGDH [197], ubiquitin carboxyl terminal hydrolase L3 (UCHL3) mediates the deubiquitination of PHGDH at Lys310 [175], and Josephin-2 deubiquitinates PHGDH in lung adenocarcinoma and HCC [ 191, 192] . This regulatory duality—degradative versus stabilizing ubiquitination—highlights PHGDH as a nodal point for modulating cancer serine metabolism, with therapeutic potential in targeting ubiquitination-associated enzymes. Furthermore, ubiquitination similarly governs PSAT1/SHMT2 stability via proteasomal or lysosomal degradation. These ubiquitination events are subject to regulation by other PTMs ( Figure 4C), which will be discussed in detail in the following sections.

Ubiquitination and SUMOylation coordinate SHMT1 nucleocytoplasmic localization and accumulation. In the S and G2/M phase of the cell cycle, SHMT1 has the lowest ubiquitination level and is transported to the nucleus after SUMO1 modification to form a dTMP synthesis complex with dihydrofolate reduction enzyme (DHFR) and thymine synthetase (TYMS) [ 194, 198] . In the same phase, K63 linkage ubiquitination of SHMT1 at Lys38 or Lys39 mediated by UBC 13 (ubiquitin conjugating enzyme 13) promotes the nuclear export of SHMT1 and increases its nuclear stability, while SUMO2/3 modification of SHMT1 at the same site mediated by UBC9 promotes its nuclear degradation [194].

##### Phosphorylation

Phosphorylation dynamically regulates PHGDH enzymatic activity through site-specific modifications. PHGDH phosphorylation dynamically regulates its enzymatic activity and stability. Protein kinase C ζ (PKCζ) phosphorylates PHGDH at Ser55, Thr57, and Thr78, with Thr57/Thr78 modifications predominating under glucose deprivation to suppress catalytic activity [185]. Glucose restriction also triggers PHGDH phosphorylation at Ser371 (p38-mediated) and Ser55 (AMPK-mediated). Ser371 phosphorylation drives nuclear translocation, while Ser55 modification redirects PHGDH activity toward malate oxidation, generating NADH (nicotinamide adenine dinucleotide) to limit nuclear NAD ^+^ levels. This suppresses PARP1-mediated c-Jun poly(ADP-ribosyl)ation, impairing transcriptional activation of oncogenic targets [186].

Phosphorylation orchestrates PHGDH/SHMT2 ubiquitination and proteasomal degradation through kinase-E3 ligase crosstalk. In CRC, GSK3β-mediated PHGDH phosphorylation at Thr497/Ser501 licenses FBXW7β-dependent ubiquitination [188]. Similarly, phosphorylation at Ser90 by mitogen-activated protein kinase 1 (MAPK1) stabilizes SHMT2 through inhibition of STIP1 homology and U-box protein 1 (STUB1)-mediated ubiquitination and subsequent proteasomal degradation [20]. Conversely, dephosphorylation of Ser90 by protein tyrosine phosphatase mitochondrial 1 (PTPMT1) promotes SHMT2 degradation. Additionally, SHMT1 has been identified as a novel substrate of p38 MAPK, though the phosphorylation site and functional consequences remain uncharacterized [199].

##### Acetylation

Acetylation directly alters enzyme conformations and intersects with ubiquitination pathways (via E3 ligases RING-finger protein 5 (RNF5), ubiquitination factor E4B (UBE4B), tripartite motif-containing protein 21 (TRIM21) or deubiquitinase USP14), establishing a post-translational regulatory axis that balances enzyme stability/activity with cancer metabolic demands. In breast cancer, PHGDH acetylation at Lys58 (mediated by Tip60) disrupts binding, inhibiting ubiquitination and degradation to promote proliferation, while SIRT2 coordinates glucose-responsive deacetylation [184]. For PSAT1, PSAT1 acetylation at Lys51 in lung adenocarcinoma triggers UBE4B-dependent K48 linked polyubiquitination and proteasomal degradation, while blocking USP14-mediated stabilization. Histone deacetylase 7 (HDAC7) deacetylates Lys51 to enhance PSAT1 stability, promoting serine metabolism [193]. Conversely, SHMT2 acetylation at Lys95 in colorectal cancer disrupts its tetrameric structure and enzymatic activity, enabling TRIM21-dependent K63-ubiquitination and lysosomal degradation to suppress serine/NADPH flux and tumor growth [195].

Furthermore, emerging evidence reveals additional regulatory acetylation events: under serine deprivation, PHGDH undergoes acetylation site switching (Lys289 to Lys21), indicative of metabolic adaptation [200], whereas gastric cancer uniquely displays SHMT2 acetylation at Lys200, underscoring tissue-specific regulation [201]. The functional consequences of these acetylation dynamics, however, remain unexplored.

##### Methylation

PRMT1-mediated arginine methylation dynamically regulates PHGDH activity and stability. PHGDH is methylated by protein arginine methyltransferase 1 (PRMT1) at Arg236, thereby enhancing catalytic activity by increasing the substrate affinity and activating serine synthesis, ameliorating oxidative stress and promoting the growth of liver cancer *in vitro* and *in vivo* [17]. Besides, PHGDH-Arg20 is modified by dimethylarginine, while Arg54, Arg268 are modified by monomethylarginine. Arg20 and Arg54 of PHGDH are located in the substrate binding domain 1 (SDB1). Methylation of Arg20 inhibits the polyubiquitination of its adjacent site Lys21 and stabilizes the active form of the enzyme, while methylation of Arg54 is critical for maintaining substrate or coenzyme affinity for PHGDH [183]. PRMT1 methylates PHGDH at Arg20/54 and increases enzyme stability or activity, thereby enhancing the serine synthesis pathway and *de novo* fatty acid synthesis. Enhanced fatty acid synthesis leads to the *de novo* synthesis of palmitate, which increases S-palmitoylation of PHGDH and sustains its enzyme activity, constituting a serine-fatty acid positive feedback loop that drives malignant traits in TNBC [183].

##### Additional modifications

Other modifications such as succinylation and fatty-acylation, have also been found to modulate SHMT2 enzymatic activity. Succinylation at Lys280 disrupts pyridoxal phosphate (PLP) binding to SHMT2, impairing active tetramer formation, while SIRT5-mediated desuccinylation restores the catalytic activity of SHMT2 to promote serine catabolism and tumorigenesis [196]. The fatty-acylation site of SHMT2 is Lys245. HDAC11 catalyzes the defattyacylation of SHMT2, promoting its translocation to late endosomes/lysosomes without altering enzymatic function [202].

### Cross-layer regulatory interactions in serine metabolism

**Figure 5:**
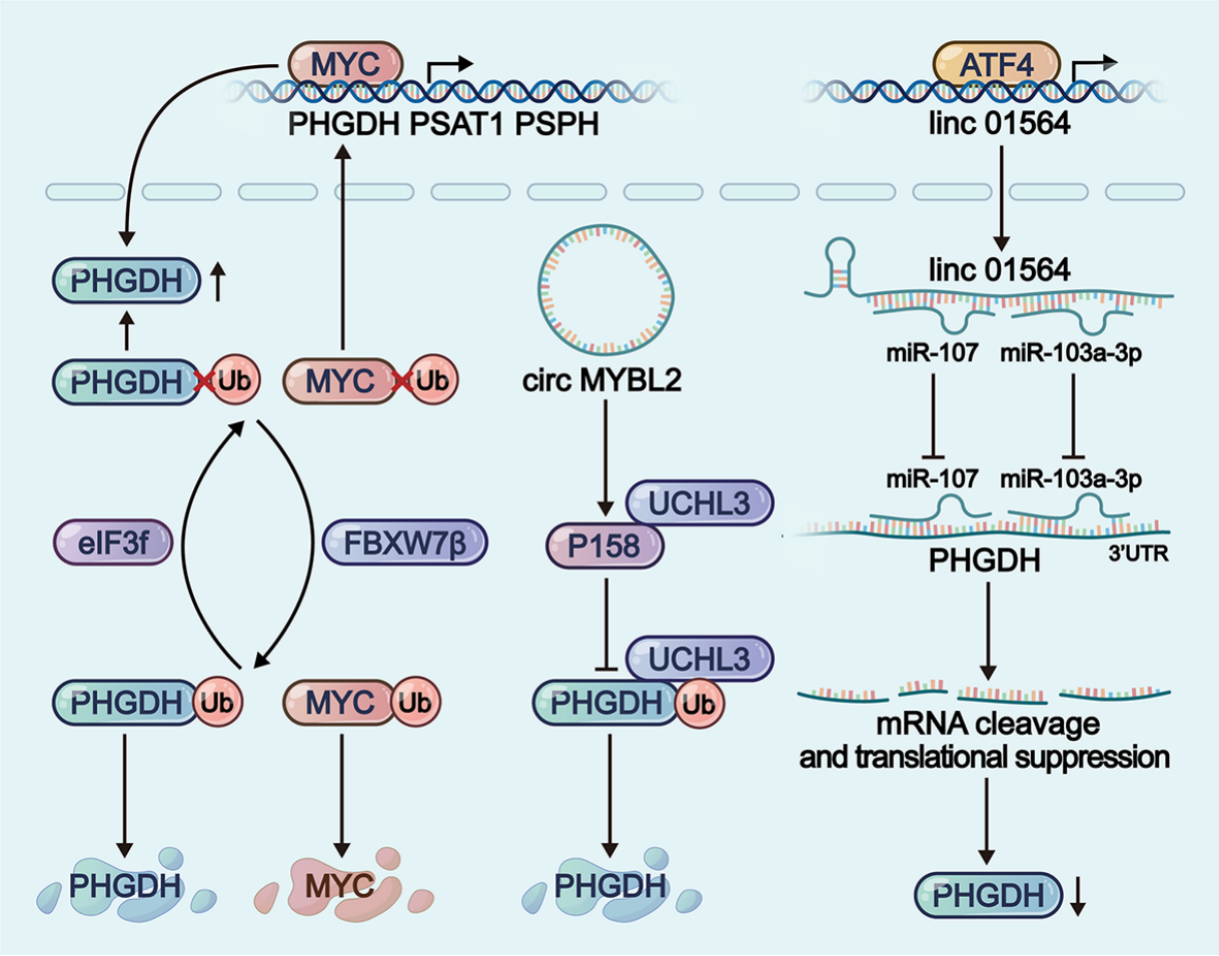
Figure 5 Cross-layer regulatory interactions in serine metabolism Serine metabolism is subject to multi-level regulation, as exemplified by the modification, RNA interference, and transcriptional mechanisms of PHGDH.

Evidence reveals that serine metabolism is regulated through cross-layer mechanisms ( Figure 5). Transcription factors can transcribe substances other than metabolic enzymes, exerting cross-layer regulatory effects. In hepatocellular carcinoma, ATF4-mediated induction of linc01564 promotes tumor survival by functioning as a ceRNA for miR-107/103a-3p [125]. Mechanistically, linc01564 sequesters miR-107/103a-3p via two complementary binding sites, thereby alleviating miRNA-mediated suppression of PHGDH [125]. Specifically, ncRNAs perform non-canonical functions by interfering with post-translational modifications. circMYBL2 encodes p185, a 185-amino-acid protein. The p185 protein inhibits PHGDH deubiquitination by competitively binding to the C1 domain of UCHL3, which results in proteasomal degradation of PHGDH and subsequent serine and glycine biosynthesis reduction [175]. Post-translational modifying enzymes act beyond metabolic enzymes to target other regulatory factors. eIF3f interacts with FBXW7β and antagonizes FBXW7β-mediated ubiquitination, resulting in the deubiquitination of PHGDH [188]. The ubiquitination of MYC is also regulated by FBXW7β and eIF3f, which further regulates the transcription of PHGDH [188].

## Higher-order Regulation of Serine Metabolic Reprogramming

**Figure 6:**
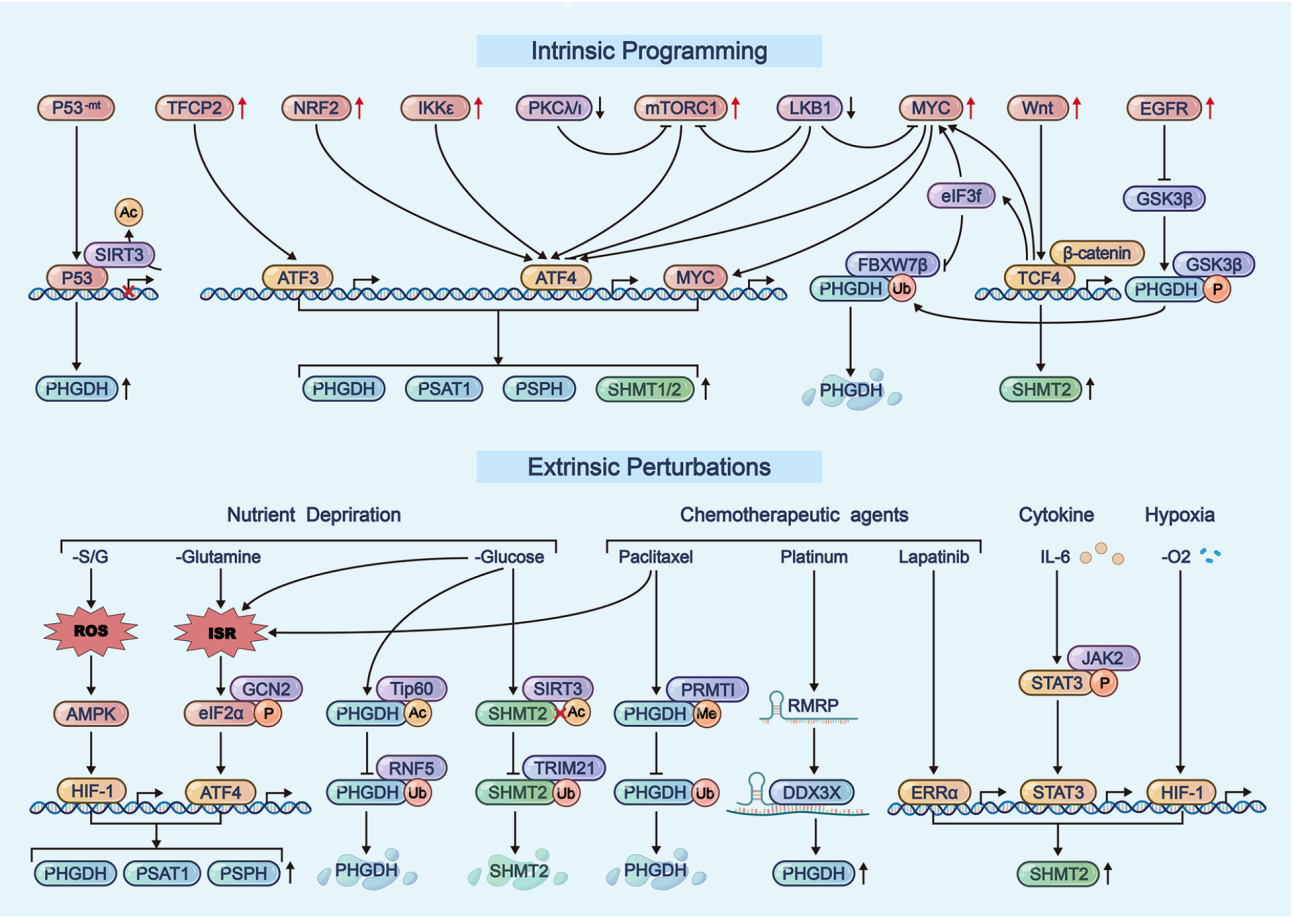
Figure 6 Higher-order regulation of serine metabolic reprogramming Serine metabolic reprogramming is orchestrated by both intrinsic factors—such as oncogenic mutations and tumor suppressor alterations—and extrinsic perturbations including nutrient availability, hypoxia, and therapeutic stress. These upstream signals converge on key regulators like ATF4 and are further modulated by post-translational modifications.

The preceding discussion outlined the hierarchical regulation of serine metabolic reprogramming. We now examine the principal upstream oncogenic regulators of this process ( Figure 6). Tumor cells frequently acquire oncogenic mutations or altered expression of tumor suppressors that modulate serine metabolism either directly or indirectly. Extracellular microenvironmental changes, including nutrient fluctuations, hypoxic conditions, chemotherapeutic agents, and cytokine signaling, further contribute to serine metabolic regulation. These cell-intrinsic and extrinsic factors function cooperatively, as exemplified by the coordinated effects of epidermal growth factor receptor (EGFR)/phosphatidylinositol-4,5-bisphosphate 3-kinase catalytic subunit alpha (PIK3CA) mutations, MYC activation, PKCζ deficiency, and nutrient stress in initiating serine metabolic reprogramming.

### Intrinsic programming

Intrinsic programming, such as oncogenic and tumor suppressor factors or the dysregulation of signaling pathways, is the initiating factor for the reprogramming of serine metabolism in tumors, representing an intrinsic feature of tumors with tumor specificity.

#### The dysregulation of oncogenes and tumor suppressors

Some oncogenic factors directly or indirectly participate in the regulation of serine metabolism. As previously mentioned, the oncogenes MYC and MYCN act as transcription factors for serine metabolic enzymes, and their amplification directly or indirectly contributes to transcription. When the tumor suppressor p53 is mutated, the competitive interaction between p53 and YY1 is lost, leading to YY1-driven PHGDH overexpression [85]. Additionally, other factors indirectly contribute to the reprogramming of serine metabolism by modulating ATF4 or ATF3, such as erythroid 2-related factor 2 (NRF2) and IκB kinase epsilon (IKKε).

Nuclear factor NRF2, a principal regulator of the cellular antioxidant response, is frequently dysregulated in NSCLC [203]. On one hand, NRF2 mutations often occur, disrupting its interaction with Kelch-like ECH-associated protein 1 (KEAP1) and thereby constitutively activating NRF2 [ 197, 204] . On the other hand, activating mutations of KRAS (Kirsten-rat sarcoma viral oncogene homolog) ^G12D^ and BRAF (The B-Raf proto-oncogene) ^V600E^ can also induce NRF2 transcription in pancreatic cancer and lung cancer [ 205, 206] . Both mutations in KRAS ^G12D^ and BRAF ^V600E^ and the knockout of *NRF2* are associated with the expression levels of serine metabolic enzymes [ 49, 76, 207] . NRF2 transcriptionally activates the synthesis of serine and glycine in NSCLC by enhancing the binding of ATF4 to the promoters of *PHGDH*, *PSAT1*, and *SHMT2* [ 76, 208] . Other studies have revealed potential underlying mechanisms. ATF4 is both a direct transcriptional target of NRF2 and can form a heterodimer with NRF2 to jointly regulate the expressions of downstream genes [76].

IκB kinase ε (IKKε), considered an oncogene in breast cancer, is overexpressed in various tumors [209]. IKKε regulates cytokine secretion through nuclear factor-kappaB (NF-κB) and interferon regulatory factor 3 (IRF3), linking inflammation and cancer. IKKε indirectly upregulates the serine biosynthesis pathway by inhibiting the activity of pyruvate dehydrogenase (PDH), thereby restricting the utilization of glucose-derived pyruvate in the TCA cycle. On the other hand, IKKε inhibits mitochondrial function, leading to ATF4 activation, which in turn drives the upregulation of SSP gene expression [78].

Mutations in Kras in tumor cells are often accompanied by the loss of liver kinase B1 (LKB1) [ 210, 211] . LKB1 is a tumor suppressor and an important metabolic regulator that modulates cellular energy balance through the activation of AMPK [212]. In KRAS ^G12D^ mutant tumor cells, the absence of LKB1 regulates the expressions of PSAT1, PSPH, SHMT1, and SHMT2 in an mTOR-dependent manner [ 213, 214] . Other studies have shown that AMPK activation leads to the suppression of mTORC1 through the activation of its negative regulator TSC2 and the inhibition of the mTORC1 subunit RAPTOR [215]. This may account for the activation of mTORC1 due to LKB1 loss. Moreover, AMPK activation suppresses MYC-driven *PHGDH* transcription, thereby impairing breast cancer proliferation [216]. This highlights the inhibitory effect of the AMPK pathway on serine metabolism.

The atypical protein kinase C (aPKC) kinase family comprises only two members: PKCζ and PKCλ/ι [217]. Extensive research and analysis have identified PKCζ as a tumor suppressor. The role of PKCλ/ι in tumors is more complex and has also been shown to exhibit tumor-suppressive effects in multiple contexts [217]. PKCλ/ι is downregulated in neuroendocrine prostate cancer (NEPC) and inhibits basal mTORC1 activity by directly phosphorylating LAMTOR2, a core component of the Ragulator complex. The loss of PKCλ/ι also activates mTORC1 and promotes serine biosynthesis via downstream ATF4 [15]. Serine synthesis leads to increased SAM, which supports cell proliferation and epigenetic changes, thereby facilitating the development of NEPC [15]. However, the specific mechanism by which mTORC1 activates ATF4 was not addressed in this study. Transcription factor cell promoter 2 (TFCP2) is a member of the TFCP2/grainyhead family of transcription factors and is considered an ideal therapeutic target and biomarker for cancer [218]. The expression of TFCP2 is upregulated in gliomas, where it interacts with ATF3 to synergistically promote the expression of PHGDH and the *de novo* synthesis of serine, thereby enhancing cell growth and sphere formation [80].

#### The aberrant activation of signaling pathways

The Wnt signaling pathway is a key regulator of development and adult tissue homeostasis and is frequently dysregulated in many cancer types [219]. Wnt signaling is a critical oncogenic pathway in CRC, modulating the expression of MYC and eIF3f through β-catenin and TCF4 signaling [188]. eIF3f antagonized FBXW7β-mediated ubiquitination of PHGDH through its deubiquitinating activity and could also deubiquitinate MYC, thereby increasing the stability of both MYC and PHGDH [188]. Another study has shown that TCF4 is also a transcription factor for SHMT2 [92], further highlighting the role of Wnt signaling in promoting serine metabolism. EGFR mutations are a major contributor to NSCLC [220]. The EGF signaling pathway is also crucial for tumors and has long been a target for human cancer therapy [220]. The EGF signaling pathway inhibits GSK3β, thereby inhibiting FBXW7β-mediated ubiquitination of PHGDH and increasing the stability of PHGDH [188].

### Extrinsic perturbations

Extrinsic modulation refers to the influence of the tumor microenvironment (TME) on tumor cells. Tumor cell survival and activities are inseparable from the TME. However, when the TME becomes unfavorable for tumor cell survival, tumor cells initiate adaptive responses, including serine metabolic reprogramming.

#### Nutrient deprivation

Nutrient deprivation, including glucose and amino acids, is a hallmark feature of the TME, primarily due to excessive nutrient consumption by tumor cells to fulfill their biosynthetic and energetic requirements [221]. Serine metabolism appears to be modulated by serine and glycine availability. As serine plays a crucial role in cancer proliferation, serine/glycine deprivation activates the SSP [222]. Serine/glycine deprivation promptly induces ROS generation, resulting in AMPK activation and subsequent AMPK-dependent stabilization and transactivation of HIF-1α. The activated HIF-1α enhances transcription of SSP enzymes, facilitating *de novo* serine and glycine biosynthesis [84]. This phenomenon presents a striking contrast to AMPK’s inhibitory role in serine metabolism, highlighting the context-dependent dual regulatory functions of AMPK. Furthermore, during serine deprivation, H3K9 methyltransferase G9A activates the expression of PHGDH and PSAT1 by regulating H3K9me1 levels around their transcription start sites [63].

Glucose and glutamine deficiency similarly constrain serine metabolism, as these nutrients supply the essential precursors 3-PG and glutamate for serine production [38]. Additionally, their deprivation may also stimulate serine metabolism. During amino acid (including serine and glutamine) or glucose deprivation, general control nonderepressible 2 (GCN2) activation induces activation of ATF4 expression, thereby initiating the serine synthesis pathway [ 14, 223, 224] . Notably, genetic ablation of ATF4 or GCN2 substantially suppressed tumor growth *in vivo* [223]. GCN2 modulates translation initiation via phosphorylation of eukaryotic initiation factor 2α (eIF2α), which promotes upstream open reading frame (uORF)-mediated ATF4 translation [225]. The GCN2/eIF2α/ATF4 axis plays a pivotal role in cellular adaptation to diverse metabolic stresses and metabolic reprogramming [226], representing a canonical activation route of the integrated stress response (ISR) [227]. Following translational upregulation, ATF4 reinforces its stability through transcriptional activation of ATF3, and cooperates with ATF3 to stimulate SSP enzyme transcription, thereby enabling *de novo* serine synthesis during serine deficiency [79].

Glucose deprivation triggers specific PTMs in serine metabolic enzymes. PHGDH phosphorylation at Ser371 drives nuclear translocation, while Ser55 phosphorylation activates malate oxidation, collectively reducing NAD⁺ levels and suppressing c-Jun-dependent transcription [186]. Concurrently, K58 acetylation protects PHGDH from RNF5-mediated ubiquitination, enhancing its stability and serine synthesis [184]. Under low glucose, SHMT2 deacetylation at K95 preserves its functional tetrameric structure and activity while evading TRIM21-dependent lysosomal degradation, thereby sustaining serine utilization and NADPH production [195]. These coordinated PTMs of PHGDH and SHMT2 under glucose restriction collectively promote tumorigenesis. These findings demonstrate that glucose scarcity reprograms serine metabolism through specific PTMs, revealing a key adaptive mechanism in cancer metabolic stress responses.

#### Hypoxia

Hypoxia is a common issue faced by solid tumors, and HIF is the principal transcriptional regulator for tumor cell adaptation to hypoxia [228]. When cells are exposed to hypoxia, HIF-1 mediates the upregulation of SHMT2 in tumor cells. Interestingly, this hypoxia-induced effect is only observed in cancer cells with MYC amplification. Since SHMT2 is a reported target gene of MYC, this suggests that MYC collaborates with HIF to induce SHMT2 expression [39]. As mentioned above, serine biosynthetic enzymes (PHGDH/PSAT1/PSPH) are downstream target of HIF-1, but whether this transcription is activated under hypoxic conditions remains to be explored.

#### Chemotherapeutic agent exposure

Chemotherapeutic agents can induce metabolic reprogramming in tumor cells as an adaptive response to drug-induced stress, contributing to chemoresistance mechanisms [229]. Estrogen-related receptor α (ERRα) can regulate the metabolic adaptation of lapatinib-resistant cancer cells. ERRα activates SHMT2 transcription by targeting the promoter region of *SHMT2*, thereby enhancing breast cancer resistance to lapatinib [92]. Furthermore, in platinum-resistant ovarian cancer, the lncRNA RMRP is upregulated and demonstrates oncogenic potential by recruiting the RNA-binding protein DDX3X to the 3′UTR of *PHGDH* mRNA, thereby enhancing PHGDH translation [176]. Paclitaxel exposure induces the ISR and activates ATF4 through the GCN2/eIF2α/ATF4 axis, upregulating SHMT2 to confer chemoresistance [230]. In paclitaxel-resistant cells, PRMT1 is responsible for the methylation of PHGDH at the Arg54 or Arg20 site. The methylation of PHGDH stabilizes 3-PG binding and inhibits polyubiquitination, thereby activating its enzymatic activity, leading to the activation of the serine synthesis pathway and the induction of protein S-palmitoylation, which confers chemoresistance to TNBC [183].

#### Immunostimulation

IL-6 is produced by multiple cell types within the tumor microenvironment and is frequently elevated in a wide range of cancers. It drives hyperactivation of the Janus kinase (JAK)/STAT3 signaling pathway, which is often associated with poor clinical prognosis [231]. In prostate cancer, IL-6 stimulates the JAK2/STAT3 canonical pathway, upregulating SHMT2 transcription, since SHMT2 is a direct transcriptional target of STAT1 [93]. Subsequently, SHMT2 activation aberrantly triggers STAT3 signaling through the PKM2 and HIF-1α/VEGF (vascular endothelial growth factor) axis, facilitating a transient shift toward anaerobic metabolism [ 47, 93] .

### Synergistic effects of intrinsic and extrinsic factors

Intrinsic programming and extrinsic perturbations are not mutually exclusive. For instance, MYC activation under conditions of glucose and glutamine deprivation not only transcriptionally upregulates SSP enzymes to directly stimulate the SSP, but also indirectly activates SSP by coordinating glycolysis and glutaminolysis. This metabolic rewiring modulates ROS levels, GSH homeostasis, apoptosis resistance, and cell cycle progression, ultimately promoting cancer cell survival and proliferation [38]. Notably, dysregulation of oncogenes or tumor suppressors alone may not suffice to induce serine metabolic reprogramming unless coupled with extrinsic stressors such as nutrient deprivation. For example, in NSCLC, activating mutations in EGFR and PIK3CA enhances ATF4 induction under amino acid stress [232]. Mechanistically, the EGFR-phosphatidylinositol-3-kinase (PI3K)-mTOR axis regulates eIF4F initiation complex assembly, synergizing with the GCN2 pathway to promote *ATF4* mRNA translation at the level of translational initiation [232]. Furthermore, PKCζ deficiency drives SSP reprogramming in glucose-deprived cancer cells [185]. Under glucose starvation, PKCζ phosphorylates and inhibits PHGDH catalytic activity, thereby suppressing serine biosynthesis. Loss of PKCζ relieves this inhibition, amplifying SSP flux and fueling intestinal tumorigenesis during metabolic stress [185].

## Role of Serine Metabolism in Tumor Immunity

Serine metabolism plays a pivotal role not only in cancer cells but also in the differentiation of immune cells within the tumor microenvironment. The M2 phenotype of tumor-associated macrophages (TAMs), which is generally associated with immunosuppressive functions, can be activated by IL-4 and the tumor microenvironment, with serine metabolism actively contributing to this activation process. Mechanistically, IL-4 and the tumor microenvironment upregulate the expression of PHGDH and PSAT1 in macrophages [ 233, 234] . The serine biosynthesis pathway mediated by PHGDH and PSAT1 facilitates the production of α-KG, a metabolite that is essential for maintaining mitochondrial function. This promotes the activation and proliferation of immunosuppressive M2 macrophages through the activation of mTORC1 signaling and Jumonji domain-containing protein D3 (JMJD3)-dependent histone modifications [ 233, 234] . In tumor-bearing mice, genetic ablation of Phgdh in macrophages suppressed TAM infiltration, promoted M1 polarization with concomitant programmed death ligand-1 (PD-L1) downregulation, and enhanced anti-tumor T cell immunity, ultimately delaying tumor growth [233]. Similarly, *Psat1* knockout in macrophages effectively inhibited M2 polarization while augmenting anti-tumor T cell responses [234].

In terms of T cells, serine metabolism exerts complex regulatory effects on their differentiation. CD4 ^+^ regulatory T (Treg) cells play a crucial role in suppressing anti-tumor immune responses. Mechanistically, serine enrichment activates mTORC1 to suppress Treg differentiation via SAMD2/forkhead box protein P3 (FoxP3) inhibition [235], whereas in the tumor microenvironment, it promotes Treg differentiation through sphinganine biosynthesis, which upregulates FoxP3 expression via the c-Fos-PD-1 axis [236]. The impact of mTORC1 on CD8 ^+^ T cell differentiation exhibits temporal specificity. During early chronic tumor stages, mTORC1 activation inhibits the differentiation of progenitor exhausted T (Tex) cells, which compromises anti-tumor immunity. In contrast, during late chronic stages, mTORC1 promotes the transition of progenitor Tex cells into effector populations [237].

In addition, serine metabolism also influences immune cell infiltration. In hepatocellular carcinoma cells, PSPH promotes monocyte/macrophage infiltration through SAM and H3K27me3/CXCL10 (C-X-C motif chemokine ligand 10), while suppressing CD8 ^+^ T lymphocyte recruitment via GSH/CCL2 (C-C motif chemokine ligand 2) [238]. In endothelial cells, PHGDH-mediated serine synthesis is upregulated, leading to excessive endothelial cell growth. Genetic PHGDH ablation inhibits this process, alleviates intratumoral hypoxia, and improves T cell infiltration into tumors [239]. Serine/glycine-free diet suppresses CRC cell growth and induces chemokine release, thereby promoting CD8 ^+^ T cell recruitment to tumor sites [240].

In therapeutic contexts, inhibition of serine metabolism enhances sensitivity to anti-PD-1 treatment. In tumor-bearing mouse models, PHGDH inhibitors, shPSPH, and serine/glycine-free diet synergized with anti-PD-1 therapy, significantly augmented anti-tumor immunity and suppressed tumor growth [ 238, 240] . These findings demonstrate the considerable potential of targeting serine metabolism for immunotherapy applications.

## Clinical Prospects of Targeting Serine Metabolism

As discussed above, targeting serine metabolism through serine metabolic enzyme inhibitors demonstrates promising antitumor efficacy. The allosteric PHGDH inhibitor disulfiram, an anti-alcoholism drug, has recently shown tumor-targeting potential [241]. Several phase I/II clinical trials have observed promising therapeutic effects of disulfiram, either alone or in combination with copper (Cu), in glioblastoma multiforme and non-small cell lung cancer patients [ 242– 244] . Mechanistic studies revealed that disulfiram inhibits PHGDH by oxidizing Cys116, thereby disrupting its active tetrameric structure and blocking serine biosynthesis [245]. Given that its antitumor mechanisms remain incompletely characterized, PHGDH-targeting activity may represent one potential contributor to its therapeutic effects. Other PHGDH inhibitors, including the allosteric inhibitor NCT-503 and covalent inhibitor PH755, have demonstrated efficacy *in vivo* [ 30, 246] , though clinical trials have not yet been initiated. Additional inhibitors such as the NAD-competitive inhibitor BI-4924 exhibit superior PHGDH inhibitory activity, but their poor plasma stability has limited *in vivo* investigation [ 247, 248] .

The antitumor efficacy of SHMT inhibitors SHIN1 and its more bioavailable derivative SHIN2 has been validated *in vivo* [ 54, 249] , though clinical trials have not yet been initiated. Antifolates including classical agents (pemetrexed, methotrexate) and the novel compound lometrexol, have also been identified as SHMT inhibitors. These folate analogs competitively bind to and inhibit SHMT [ 250– 252] . While pemetrexed and methotrexate are already clinically utilized in cancer treatment [253], lometrexol completed a phase 2 trial for non-small cell lung cancer without published results (NCT00033722). The antidepressant sertraline was found to bind to and inhibit mitochondrial SHMT2, demonstrating synergistic effects with radiotherapy in suppressing NSCLC tumor growth, reducing cancer stemness, and enhancing antitumor immunity [254]. Other serine metabolic enzymes also show promising therapeutic targets, as evidenced by PSAT1 inhibitor AOA’s antitumor effects, though human trials remain pending. These findings collectively outline both current clinical applications and future therapeutic potential in targeting serine metabolism.

While specific inhibitors targeting serine metabolic enzymes have not yet entered clinical trials, with only some non-specific inhibitors being discovered to have inhibitory effects post clinical application, specific inhibitors targeting transcriptional regulators or upstream pathways of serine metabolism have already achieved clinical utility or are under investigation. mTORC1 inhibitors ( *e.g*., everolimus, temsirolimus) have demonstrated efficacy in renal cell carcinoma and breast cancer, though overcoming monotherapy limitations remains challenging [255]. The first clinical trial results of an MYC inhibitor were recently reported, with OMO-103 showing favorable safety and preliminary antitumor activity in a phase I trial for advanced solid tumors. Eight out of twelve patients achieved stable disease, including one pancreatic cancer patient exhibiting a 49% reduction in total tumor volume [256]. These clinical applications and investigations of upstream pathway inhibitors targeting serine metabolism provide novel therapeutic strategies for cancer treatment.

Therapeutic combinations targeting serine metabolism have demonstrated promising synergistic effects in preclinical studies. Both 5-FU and the SHMT2 inhibitor SHIN1 suppress dTMP synthesis, and their co-administration synergistically depletes nucleotide precursors by activating p53 signaling to induce G1-phase cell cycle arrest and DNA damage, thereby enhancing cytotoxicity against gastric cancer cells and overcoming 5-FU resistance [257]. Serine/glycine-free diets significantly inhibited tumor growth in chemotherapy-resistant patient-derived xenograft models, with cisplatin combination further improving efficacy. Mechanistically, serine metabolism inhibition attenuates DNA repair capacity in platinum-resistant cells by reducing nucleotide synthesis and NAD ^+^ availability, consequently potentiating cisplatin-induced DNA damage [258]. PHGDH inhibitors (NCT-503 and CBR-5884) also reduce cisplatin sensitivity in gastric cancer cells by decreasing H3K4 trimethylation, leading to chromatin compaction and impaired DNA damage repair [259]. In addition, some drugs ( *e.g*., sorafenib) induce resistance associated with SSP upregulation. Combination treatment with NCT-503 and Sorafenib significantly outperforms monotherapy, completely halting the growth of HCC *in vivo* [260]. Given the critical role of serine metabolism in tumor immunity, its targeting enhances anti-PD-1 efficacy as previously discussed. These findings collectively suggest that combining serine metabolism inhibitors with conventional chemotherapeutics or immunotherapies could be a novel synergistic treatment strategy by sensitizing resistant tumors.

## Prospects

Serine metabolic enzymes—encompassing anabolic enzymes (PHGDH, PSAT1, PSPH) and catabolic enzymes (SHMT1, SHMT2) are commonly upregulated in cancers to fuel rapid proliferation. This review systematically classifies their regulatory mechanisms into three tiers: transcriptional regulation, post-transcriptional regulation, and post-translational modifications. While DNA copy number alterations and translational regulation occasionally occur in specific malignancies, their limited prevalence and mechanistic ambiguity preclude comprehensive discussion here. Together, we present an overview of the common and well-studied regulatory mechanisms in an attempt to shed new light on cancer treatment.

Notably, multilayered crosstalk exists across these regulatory axes. For example, MYC directly transcribes PHGDH and PSAT1, while also indirectly modulating their expression through ATF4. Conversely, TCF4 (the transcription factor of SHMT2) activates MYC, forming a feedforward loop. The most recognized transcription factor ATF4, in addition to directly transcribing serine metabolic enzymes, can also upregulate PHGDH by transcribing linc01564. ATF4 is also a direct target of KDM4C (histone demethylase of PHGDH and PSAT1), leading to its transcriptional activation. And ATF3, in addition to being transcribed by ATF4, binds to MDM2 (the E3 ubiquitin ligase of p53) to promote the transcription of *PSAT1*. Such complexity suggests an overarching regulatory network coordinating serine metabolism—a hypothesis warranting rigorous exploration to unravel metabolic plasticity in cancer.

Equally critical are self-reinforcing feedback loops that amplify oncogenic signaling. Blocking these positive feedback pathways may be more effective for therapies targeting serine metabolism. For example, the positive feedback pathway of β-catenin/TCF4/SHMT2/β-catenin in CRC, STAT3/SHMT2/PKM2/STAT3 in prostate cancer, SAM/H3K27me3/MYC/SAM, and the mutual enhancement of *de novo* fatty acid synthesis and S-palmitoylation of PHGDH. These positive feedback pathways need exceptional attention in the study of serine metabolic enzymes, as therapeutic disruption of these circuits may prove superior to targeting individual nodes.

While serine metabolism has been the subject of scientific investigation for decades as a pivotal branch of amino acid metabolism, systematic investigation of its enzymatic machinery gained substantial momentum in the 21st century. Despite the current progress in serine metabolism, there are still some important questions remain to be elucidated. For example, revealing the preference of different types of cells for serine metabolism in the tumor microenvironment; exploring how different types of post-translational modifications cooperate to regulate the function of serine metabolizing enzymes; clarifying how the cytosolic and mitochondrial SHMT cooperate to regulate serine catabolism under different physiological conditions; developing specific inhibitors targeting serine metabolic enzymes and validating their tumor suppressive effects. Unveiling these problems will be helpful for us to understand the regulatory mechanisms of tumor serine metabolism and develop new antitumor drugs by targeting serine metabolism.
